# Cholinergic Control of GnRH Neuron Physiology and Luteinizing Hormone Secretion in Male Mice: Involvement of ACh/GABA Cotransmission

**DOI:** 10.1523/JNEUROSCI.1780-23.2024

**Published:** 2024-02-06

**Authors:** Csaba Vastagh, Imre Farkas, Veronika Csillag, Masahiko Watanabe, Imre Kalló, Zsolt Liposits

**Affiliations:** ^1^Laboratory of Endocrine Neurobiology, HUN-REN Institute of Experimental Medicine, Budapest H-1083, Hungary; ^2^Department of Anatomy, Hokkaido University School of Medicine, Sapporo 060-8638, Japan

**Keywords:** acetylcholine, GABA, GnRH, optogenetics, patch clamp, pharmacogenetics

## Abstract

Gonadotropin-releasing hormone (GnRH)-synthesizing neurons orchestrate reproduction centrally. Early studies have proposed the contribution of acetylcholine (ACh) to hypothalamic control of reproduction, although the causal mechanisms have not been clarified. Here, we report that in vivo pharmacogenetic activation of the cholinergic system increased the secretion of luteinizing hormone (LH) in orchidectomized mice. 3DISCO immunocytochemistry and electron microscopy revealed the innervation of GnRH neurons by cholinergic axons. Retrograde viral labeling initiated from GnRH-Cre neurons identified the medial septum and the diagonal band of Broca as exclusive sites of origin for cholinergic afferents of GnRH neurons. In acute brain slices, ACh and carbachol evoked a biphasic effect on the firing rate in GnRH neurons, first increasing and then diminishing it. In the presence of tetrodotoxin, carbachol induced an inward current, followed by a decline in the frequency of miniature postsynaptic currents (mPSCs), indicating a direct influence on GnRH cells. RT-PCR and whole-cell patch-clamp studies revealed that GnRH neurons expressed both nicotinic (α4β2, α3β4, and α7) and muscarinic (M1–M5) AChRs. The nicotinic AChRs contributed to the nicotine-elicited inward current and the rise in firing rate. Muscarine via M1 and M3 receptors increased, while via M2 and M4 reduced the frequency of both mPSCs and firing. Optogenetic activation of channelrhodopsin-2–tagged cholinergic axons modified GnRH neuronal activity and evoked cotransmission of ACh and GABA from a subpopulation of boutons. These findings confirm that the central cholinergic system regulates GnRH neurons and activates the pituitary–gonadal axis via ACh and ACh/GABA neurotransmissions in male mice.

## Significance Statement

Cholinergic drugs influence reproduction centrally, although the exact neuronal targets and regulatory mechanisms remain unsettled. We found that pharmacogenetic activation of the cholinergic system in vivo evoked an augmented LH release in male mice. The study also identified cholinergic cell groups in the mouse forebrain that innervate gonadotropin-releasing hormone (GnRH) neurons, the main hypothalamic regulators of reproduction. We also determined the subtypes of nicotinic and muscarinic receptors involved in neuronal information transfer and explored how their ligands affect the electrophysiological activity of GnRH neurons. A subset of cholinergic neurons was found to cotransmit GABA which excites GnRH cells via GABA_A_ receptors. The findings suggest cholinergic regulation of the GnRH system activating the pituitary–gonadal axis in adult male mice.

## Introduction

In vertebrates, the success of reproduction depends on the integrity of the hypothalamo–pituitary–gonadal axis and the availability of the master molecule, gonadotropin-releasing hormone (GnRH; Knobil, 1988). GnRH is synthesized in neurons of olfactory placode origin (Wray et al., 1989), which migrate through the basal forebrain and settle down at species-specific anatomical locations (Silverman et al., 1979; Merchenthaler et al., 1980; Liposits et al., 1984). Via their hypophysiotropic axon projections, the cells secrete GnRH in a pulsatile manner into the hypophysial portal circulation (Carmel et al., 1976) to control the proper gonadotroph hormone output of the anterior pituitary. The loosely organized GnRH cell population receives neuronal inputs from >50 different brain regions (Boehm et al., 2005) and processes and integrates information on circulating gonadal hormones (Christian and Moenter, 2010; Farkas et al., 2018) and a wide range of peripheral metabolic signals (Farkas et al., 2013, 2016; Csillag et al., 2019; Balint et al., 2020). Failure of cell migration, disturbances in sensing and processing peripheral hormone signals, or inability to respond properly to neurotransmitters and neuropeptides released from their synaptic afferents result in GnRH neuron and HPG axis dysfunctions or infertility (Pitteloud et al., 2006, 2007). These pathological events interfere with the maintenance of species; therefore, the understanding of the diverse regulatory mechanisms of GnRH neurons at the molecular and cellular levels has been essential.

Most of the classic neurotransmitter systems, including glutamate (Iremonger et al., 2010) and GABA (DeFazio et al., 2002), have been shown to regulate reproduction centrally via synaptic channels in GnRH neurons. Acetylcholine (ACh) is a highly potent neurotransmitter used widely for communication in both the central and peripheral nervous systems. Its effects are executed via nicotinic (nAChR; Albuquerque et al., 2009) and muscarinic ACh receptor (mAChR; Caulfield and Birdsall, 1998) subtypes that are differentially expressed in diverse neuron phenotypes. Cholinergic signaling has been proposed to control reproduction centrally. Early in vivo studies have shown that nicotine delays the surge release of luteinizing hormone (LH; Blake et al., 1972), inhibits the progesterone-advanced LH surge in proestrus (Kanematsu and Sawyer, 1973), blocks the LH secretion induced by opioid receptor antagonists (Hodson et al., 1997), and decreases the pulsatile secretion of LH (Sano et al., 1999). Under in vitro conditions, the natural ligand ACh stimulated the release of GnRH from hypothalamic fragments and the secretion of LH (Fiorindo and Martini, 1975). The involvement of muscarinic mechanisms has also been shown since selective muscarine receptor antagonists stimulated the release of GnRH from the median eminence of the rat (Koren et al., 1992). The use of immortalized GnRH neurons (GT1–7 cells) allowed the characterization of some molecular mechanisms involved in the cholinergic modulation of GnRH neurons. These cells express functional, high-affinity α4β2 and α7 subtypes of nAChRs, and the activation of the α-bungarotoxin–sensitive α7 subclass by nicotine exerts an inhibitory effect on GnRH release (Messi et al., 2018). A biphasic action of ACh on GnRH release was seen in perifused hypothalamic cells and GT1–7 neurons, whereas nicotine alone increased and muscarine decreased the release of the neurohormone (Krsmanovic et al., 1998). While nicotinic and muscarinic M1 receptors exert stimulatory effects, activation of muscarinic M2 receptors inhibits GnRH release (Krsmanovic et al., 1998). Transcriptome profiling of rodent GnRH neurons and GT1–7 cells has confirmed the expression of certain nicotinic and muscarinic receptors (Todman et al., 2005; Varju et al., 2009; Arai et al., 2017), supporting the view of a direct action of cholinergic drugs upon GnRH neurons. It has been shown recently that murine GnRH neurons grown in nasal explants receive regulatory ACh neurotransmission (Shostak et al., 2023). Although these findings unequivocally imply that the central cholinergic system is capable of modulating GnRH neurons, the molecular, cellular, and physiological features of this regulatory channel are largely unidentified in adult mice.

Therefore, the present study was undertaken in adult male mice to (1) measure the impact of in vivo chemogenetic activation of cholinergic neurons on LH secretion; (2) explore the putative networking of the central cholinergic system with hypophysiotropic GnRH neurons; (3) elucidate the source(s) of ACh involved in neurotransmission; (4) reveal the type(s) of cholinergic receptors expressed in GnRH neurons, and (5) characterize the effects of AChR ligands on the physiology of GnRH cells.

## Materials and Methods

### Experimental design and statistical analysis

#### Pharmacogenetics

To test the putative regulatory role of the central cholinergic system on LH release, we performed selective chemogenetic activation of cholinergic neurons by CNO (clozapine N-oxide) in orchidectomized, choline acetyltransferase (Chat)-G_q_ (hM3Dq) designer receptor exclusively activated by designer drug (DREADD) mice in split-plot design. Serial, whole blood samples were collected from vehicle- and CNO-treated mice and their LH levels determined by an ultrasensitive ELISA technique. ANCOVA was performed to estimate the effect size of the CNO treatment on the LH values [mean and basal serum levels, area under curve (AUC), amplitude, and frequency], implementing between-group (vehicle vs CNO) and within-subject (pre- vs post-treatment time) variables using the R software environment (R Core Team, 2023). When the post-treatment LH values for the two groups were statistically different (*p *< 0.05) after controlling for the pretreatment LH level as a covariate (Crager, 1987), the adjusted group means (estimated marginal means) were calculated and Tukey’s pairwise comparisons were conducted as a post hoc test.

#### Neurochemical studies on networking

The networking of the central cholinergic system with GnRH neurons was studied then by a high-throughput double immunofluorescent approach based on the 3DISCO clearing technique. Thick brain slices of adult GnRH-GPF male mice allowed to maintain the structural integrity of the region of interest for the purpose of image reconstruction via confocal microscopy. The distribution of vesicular acetylcholine transporter (VAChT)-immunoreactive (IR) axon juxtapositions on GnRH dendrites and somata was analyzed, and the ratio of innervated GnRH neurons was determined.

Two-sample *t*-tests were used to compare the number of VAChT-IR axon juxtaposition on somata and dendrites of GnRH neurons as well as the number of innervated and noninnervated GnRH somata. The level of significance was set at *p* < 0.05.

Immunocytochemical triple labeling was used for tracing expression of the GABAergic marker, vesicular GABA transporter (VGAT) in cholinergic axons innervating GnRH neurons. Juxtaposition of VAChT-IR varicosities on GnRH neurons was further characterized at the ultrastructural level by a double-labeling technique identifying VAChT-IR (by nickel-DAB) and GnRH-IR (by silver-intensified colloidal gold particles) profiles. Contacts were examined through a series of ultrathin sections.

#### GnRH-Cre neuron-dependent retrograde rabies virus labeling

To identify the location/s/ of ChAT-IR neurons that monosynaptically innervate GnRH neurons, the septo-preoptic area of GnRH-Cre mice was infected in two steps with genetically altered helper and rabies viruses (Wall et al., 2010; Roelofs et al., 2021). Helper virus-infected GnRH-Cre neurons expressing avian tumor virus receptor A (TVA) receptor and G-proteins became targets of the modified, envelope protein A (EnvA)-coated, G-deleted rabies virus (RVdG) which then labeled the monosynaptically connected afferent neurons with their fluorescent transgene. Immunohistochemical detection of the fluorescent protein products of transgenes was combined with ChAT immunolabeling to identify the cholinergic phenotype and location of the afferent neurons.

#### Expression profiling of cholinergic receptors in GnRH neurons

The question whether GnRH neurons express cholinergic receptors was studied by TaqMan PCR technique. The relative gene expression (mRNA) levels of cholinergic muscarinic receptors and subunits of nicotinic receptors were calculated in pooled GnRH cytoplasmic samples to determine whether GnRH neurons are direct targets of cholinergic neurotransmission.

#### Whole-cell patch-clamp studies

Slice electrophysiology was used to elucidate the specific role of different mAChR and nAChR in regulation of GnRH neurons. Inward membrane currents and postsynaptic currents (PSCs) in GnRH neurons were recorded in whole-cell voltage-clamp mode at −70 mV holding potential. Various selective and nonselective AChR agonists were applied in a single bolus pipetted into the measurement chamber onto the acute brain slice, and changes in the membrane current or in the frequency of miniature postsynaptic currents (mPSCs) were measured. These measurements were repeated in the presence of various cocktails of selective and nonselective AChR antagonists to demonstrate the role of the diverse nAChR and/or mAChR in the observed effects.

Changes in the firing rate of GnRH neurons resulting from activation of AChRs were proven in whole-cell current-clamp mode at 0 pA holding current. AChR agonists (ACh, carbachol, or muscarine) were applied in a single bolus pipetted directly onto the brain slice and then the change in firing rate was examined. These measurements were then repeated in the presence of various cocktails of AChR antagonists.

#### In vitro optogenetics

Involvement of the endogenous ACh release from the axon terminals contacting GnRH neurons was investigated by optogenetic examinations in brain slices from Chat-Cre-ChR2-GnRH-GFP mice, expressing channelrhodopsin in axon boutons containing ACh. LED illumination of the acute brain slices at 470 nm and 5 Hz was used to release ACh from these boutons, and changes in the firing rate or frequency of mPSCs were recorded. The measurements were repeated in the presence of mecamylamine/atropine.

Frequency-dependent GABA versus ACh synaptic release to GnRH neurons from the channelrhodopsin-expressing ACh-containing axon terminals was revealed in brain slices by optogenetics from triple-transgenic mice. LED illumination of two frequencies (0.2 Hz vs 5 Hz) was used to evoke GABAergic and/or cholinergic PSCs. Then the measurements were repeated in the presence of the GABA_A_-R antagonist picrotoxin and mecamylamine/atropine, respectively.

Electrophysiological recordings were stored and analyzed offline. Event detection was performed using the Clampfit module of the pClamp 10.4 software (Molecular Devices). Firing rates and mPSC frequencies were calculated as the number of APs or mPSCs divided by the length of the corresponding time. The mean values of the control and treated periods of the recording were calculated from these frequency values. Group data were expressed as mean ± standard error of mean (SEM). Two-tailed Student’s *t*-test, or one- or two-way ANOVA, was applied for comparison of groups followed by Tukey’s post hoc test on the frequency and on the current amplitude data, and the differences were considered significant at *p* < 0.05. The detailed data of statistical analyses are shown in extended data figures linked with Figures 6–11.

### Animals

Adult wild-type, and transgenic mice on the C57Bl/6 J or Bl6Fx background were used from local colonies bred at the Medical Gene Technology Unit of the Institute of Experimental Medicine (IEM). All animals were housed in a light- (12 h light/dark cycle, lights on at 06:00 h) and temperature-controlled (22 ± 2°C) environment, with *ad libitum* access to standard food and tap water. All surgical interventions and perfusions were carried out under deep anesthesia by intraperitoneal injections of a cocktail of ketamine (25 mg/kg body weight) and xylazine (5 mg/kg body weight).

Animal usage in the different experiments is summarized in Table 1.

**Table 1.** A summary of animal use in the various experiments

	Detection by	Aims: demonstrating	Experimental TG animals	*N*	Control animals	*N*
Pharmacogenetics	LH ELISA	Effects of cholinergic activation upon LH secretion	ChAT-G_q_ (hM3Dg) DREADD	13 ♂	N/A	
Neuroanatomy	IF–double and triple labeling and 3DISCO technique–confocal microscopy	Connection sites of ChAT-IR fibers onto GnRH neurons VAChT and VGAT colocalization in GnRH afferents Specific expression of ChR2 or hM3Dq DREADD in ChAT-IR neurons	GnRH-GFP	6 ♂ 4 ♂ 6 ♂	C57Bl/6 J	15 ♂
	IHC–double labeling–electron microscopy	Connection type(s) of ChAT-IR fibers on GnRH neurons	GnRH-GFP	10 ♂	N/A	
	IHC–retrograde tract tracing	Primary cholinergic afferents of GnRH neurons	GnRH-Cre	15 ♂	C57Bl/6 J	13 ♂
RT-PCR	TaqMan assays	Transcripts of ACh-binding receptor subunits in GnRH neurons	GnRH-GFP	7 ♂	N/A	
Electrophysiology	Whole-cell patch clamp	Effects of AChR agonists and antagonist in GnRH neurons	GnRH-GFP	98 ♂	N/A	
	In vitro optogenetics	Effects of cholinergic activation upon GnRH neurons	Chat-Cre/ChR2/GnRH-GFP	32 ♂	N/A	

#### Transgenic mouse lines and characterizations

##### The GnRH-GFP mouse line

The GnRH-GFP transgenic mouse line was generated to facilitate the study of hypothalamic GnRH neurons in which the green fluorescent protein (GFP) is genetically targeted to these cells (Suter et al., 2000b). The expression of GFP was detected in 84–94% of GnRH immune-positive neurons. This mouse line has been extensively used in studies aimed at electrophysiological characterization (Suter et al., 2000a; Christian et al., 2005; Pielecka-Fortuna et al., 2008; Farkas et al., 2010, 2016) and cell type-specific genomic analysis of the GnRH neurons (Todman et al., 2005; Vastagh et al., 2015, 2016).

##### The Chat-Cre mouse line

Chat-IRES-Cre knock-in mice that express Cre recombinase in cholinergic neurons were purchased from the Jackson Laboratory (JAX stock 006410). The line was rederived at the Medical Technology Unit of the IEM, Hungary (Van Keuren and Saunders, 2004). Stock 006410 animals were bred to the ROSA26::FLPe strain to facilitate the removal of the *frt*-neo cassette between the Cre and SV40 poly(A) to exclude ectopic Cre expression. The resulting animals with the Chat-Cre-Δneo knock-in allele were bred together, while the ROSA26::FLPe was removed, to establish the Chat/cre_Δneo//Gt(ROSA)26Sor/CAG/LSL mouse line.

##### The Chat-G_q_ (hM3Dq) DREADD mouse line

**Figure 1.**
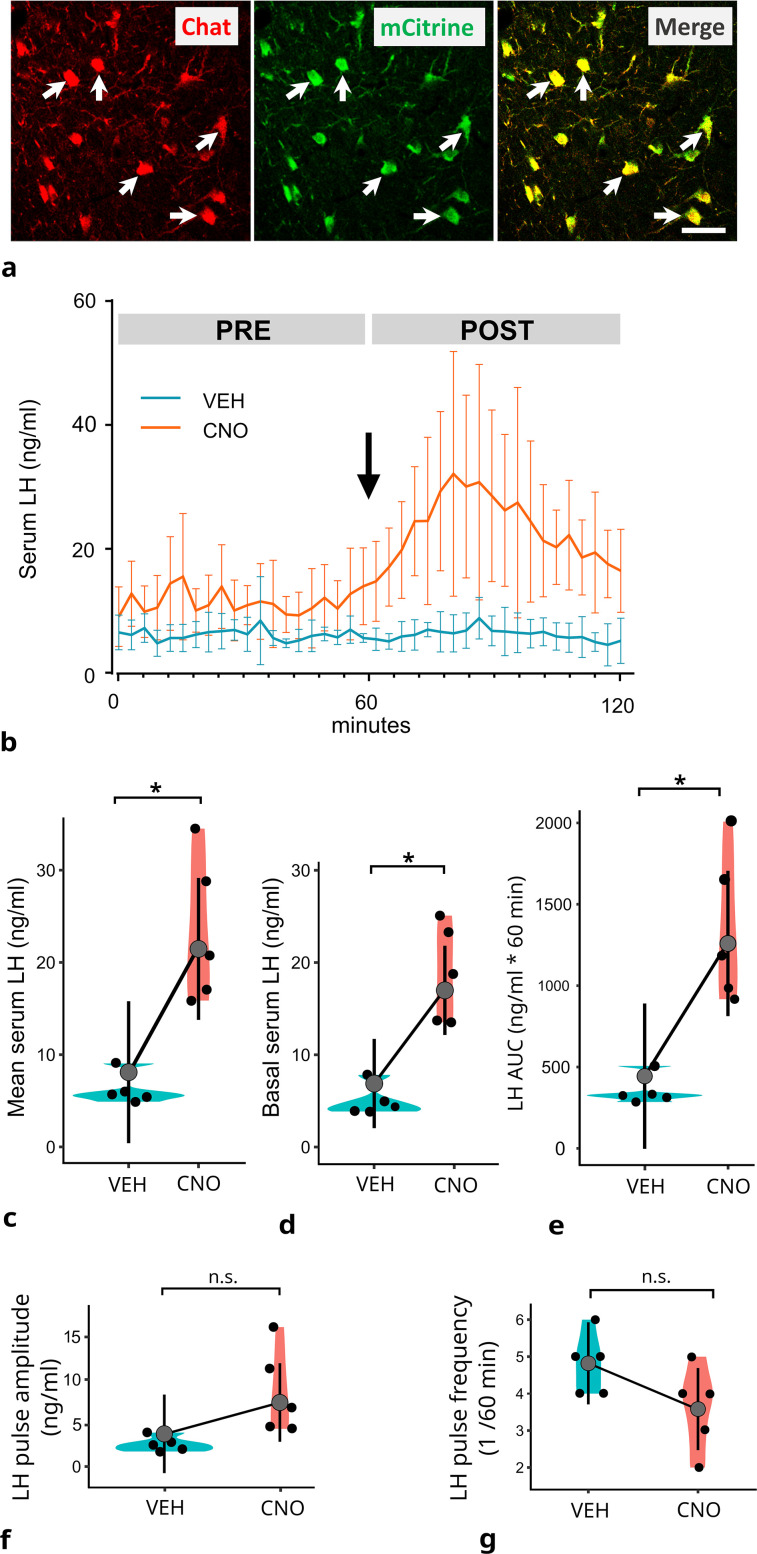
Effect of CNO treatment on serum LH level in orchidectomized Chat-Cre-G_q_ DREADD mice. ***a***, Expression of G_q_ (hM3Dq) DREADD receptor in cholinergic neurons of the MS in the Chat-G_q_ (hM3Dq) DREADD mouse. Cholinergic neurons immunolabeled for choline acetyltransferase (Chat, red) are immunopositive for mCitrine (green), the reporter protein identifying the expression sites of the DREADD receptor. Merged image demonstrates the overlap (yellow) of Chat-IR and mCitrine-IR. Arrows point to double-labeled neurons. Scale bar, 25 µm. ***b***, Effects of vehicle (VEH, blue bars) and CNO (red bars) treatments upon serum LH levels. Blood samples were collected every 3 min over a 2 h period. The arrow indicates the time of vehicle or CNO administration. PRE, pretreatment period; POST, post-treatment period. The plot shows the mean LH levels and 95% CI per sampling time, measured by ELISA. ***c***, Adjusted mean serum LH levels in vehicle (VEH) and CNO-treated (CNO) groups. The mean LH level was significantly higher in the CNO-treated group (21.5 ± 7.7) compared with that in the VEH group (8.10 ± 3.26), *p < 0.05. ***d***, Basal LH level was significantly higher in the CNO-treated group (16.9 ± 2.03) compared with that in vehicle group (6.84 ± 2.03), *p < 0.05). ***e***, The adjusted LH AUC means of the post-treatment period were higher in the CNO group (1,269 ± 189, 95 ng/ml * 60 min) compared with that in the vehicle-treated group (444 ± 189, 95 ng/ml * 60 min; *p < 0.05). The CNO treatment had no significant effect on the amplitude (***f***) or frequency of LH pulses (***g***). LH peaks and their frequencies were detected using the PULSAR application. From ***c*** to ***g***, gray dots indicate adjusted mean data; vertical lines indicate ± margin of errors at 95% CI, df = 7; black dots show the mean data of individual mice measured in the treatment period. *p*, adjusted *p*-values, Tukey’s post hoc; CI, confidence interval. The adjusted mean ± SEM values (*n* = 5) were calculated throughout the 60 min sampling period. Details of the statistical analysis are provided in Extended Data Figure 1-1.

Chat/Cre_Δneo mice were crossed with B6N;129-Tg(CAG-CHRM3*,-mCitrine)1Ute/J mice (Jackson Laboratory, JAX stock #026220) to express designer receptors (DREADD) signaling via G_q_ (hM3Dq; Armbruster et al., 2007) in cholinergic neurons. This mouse line was used for pharmacogenetics. Its immunocytochemical characterization revealed mCitrine expression in Chat-IR neurons of the brain. The established new mouse line was used for in vivo chemogenetic activation of the cholinergic system, including those subsets of cholinergic neurons that modulate the hypothalamic control mechanisms of reproduction. The immunocytochemical phenotyping of the transgenic mouse strain confirmed the cholinergic neuron-specific expression of the excitatory hM3Dq DREADD receptor (Fig. 1*a*).

10.1523/JNEUROSCI.1780-23.2024.f1-1 Figure 1-1 Estimated marginal means and contrast of serum LH, LH AUC and peak LH frequency. One-way ANCOVA followed by Tukey's post-test. Download Figure 1-1, DOCX file.

##### The Chat-Cre-ChR2 mouse line

Chat-Cre-Δneo male mice were crossed with channelrhodopsin-2 (ChR2) reporter mice [Ai32(RCL-ChR2(H134R)/eYFP), JAX stock 012569], for the purpose of optogenetics and morphological studies (Madisen et al., 2012). Using an eyfp reference gene assay mix, the copy number of the Cre and enhanced yellow fluorescent protein (eYFP) transgenes in the offspring was measured. Animals homozygous for Cre and eYFP were selected for morphological experiments, as well as for cross-breeding with the GnRH-GFP mouse line. The cholinergic neuron-specific expression of ChR2 was confirmed by double immunofluorescence staining for the choline acetyltransferase (ChAT) enzyme and eYFP (Fig. 11*a*).

##### The Chat-Cre-ChR2-GnRH-GFP mouse line

The triple-transgenic, Chat-Cre-ChR2-GnRH-GFP mouse line was established for the purpose of the optogenetic study using in vitro acute brain slice electrophysiology. In this experimental design, blue LED (470 nm) light stimulation of the ChR2-expressing axons of cholinergic neurons distributed in the medial preoptic area (mPOA) and whole-cell patch recording from GFP-expressing GnRH neurons were performed simultaneously. To carry out this goal, we crossed homozygous male Chat-Cre-ChR2 mice with female GnRH-GFP mice. The presence of the GnRH-GFP transgene in the offspring of the first generation was confirmed using quantitative real-time PCR (qRT-PCR); the relative copy number of the gfp transgene (3 vs 2) was distinguishable using this method. The immunocytochemical phenotyping confirmed that GnRH neurons receive innervation from ChR2-expressing cholinergic neurons.

##### The GnRH-Cre mouse line

Transgenic mice expressing Cre recombinase in GnRH neurons (GnRH-Cre) were used to determine brain regions that host cholinergic cells innervating GnRH neurons. The generation and characterization of this mouse line were published elsewhere (Yoon et al., 2005). Briefly, a 212 kb bacterial artificial chromosome (BAC) holding the entire GnRH coding sequence was modified: a Cre recombinase-poly(A) cassette was inserted into the ATG of the first exon of the GnRH gene, resulting in a high (over 96%) colocalization rate of the anti-Cre and anti-GnRH IRs (Yoon et al., 2005).

### Studies on cholinergic modulation of LH secretion

#### Pharmacogenetics

Transgenic male (*n* = 13) mice expressing mCitrine and activating (hM3Dq) DREADDs [Chat/cre_Δneo//GT(ROSA)26Sor_CAG/LSL_CHRM3_mCitrine (TgTm): Bl6Fx] in cholinergic neurons were used. Mice were bilaterally orchidectomized (ORX) under ketamine–xylazine anesthesia. After a week of recovery, all mice were handled and trained for tail-tip bleeding every day (McCosh et al., 2018), for at least 28 d. A week before blood collection, animals were also trained every day to lick off a small bolus of low sugar containing peanut cream (∼20 mg) mixed with 1 µl DMSO after stroking the tail (Mamgain et al., 2021).

After clipping the tip of the tail (1–2 mm), mice were placed on a flat surface every 3 min and allowed to roam freely before 3 µl of blood was collected from the tail wound. The first 10 blood samples were discarded to eliminate samples deriving from the after-cut period potentially affected by stress. The subsequent blood samples were immediately mixed with 57 µl of 0.1 M phosphate-buffered saline (PBS; Invitrogen) containing 0.05% Tween 20 and 0.2% bovine serum albumin (BSA; Jackson ImmunoResearch) and preserved in prediluted form in 60 µl aliquots on ice during blood collection and then stored at −20°C until LH assay. Samples for LH measurements were collected for 180 min, which generated 60 aliquots per mouse. After collecting the 29th blood sample, animals either received vehicle (1 µl DMSO; *n* = 5) or the DREADD ligand CNO (Tocris; 0.1 mg/µl DMSO; 5 mg/kg body weight; *n* = 5) in a single bolus of peanut cream. For LH measurement, samples collected over 60 min before and after treatments were used.

#### Ultrasensitive LH measurement and pulse analysis

The pattern of LH secretion was monitored and measured by an ultrasensitive ELISA technique according to the protocol published earlier (Steyn et al., 2013). LH concentrations were calculated by interpolating OD values from 0.00195 to 4 ng/ml onto a standard curve using a four-parameter logistic (4PL) curve (*R*^2^ = 1). Assay sensitivity—the lowest concentration level that can be determined to be statistically different from a blank at a 99% confidence level—was determined to be 0.583 ng/ml. The average intra- and interassay coefficient of variations were 3.54 and 8.61%, respectively.

The identification of LH pulses was performed using an adapted version of the PULSAR algorithm (Merriam and Wachter, 1982; Porteous et al., 2021) with the specific parameters that were recently published to optimize pulse detection in GDX mice (Porteous et al., 2021): smoothing, 0.7; G1 = 2.2; G2 = 2.7; G3 = 1.9; G4 = 1.5, G5 = 1.2; peak split, 2.5.

Average values for mean LH, basal LH, pulse amplitude, and pulse frequency were calculated for each animal in separate 60 min pre- and post-treatment periods. Mean and basal LH levels were calculated by averaging either all measured LH values or the 10 lowest LH values, respectively, in the 60 min sampling period before and after CNO or vehicle administration for each animal (Czieselsky et al., 2016). The pulse amplitude was determined by subtracting the pulse peak from the preceding nadir and then averaging this value over the sampling period for each animal (Kreisman et al., 2022). The pulse frequency was quantified as the total count of LH pulses occurring within the 60 min sampling period.

### Neuroanatomical studies on cholinergic innervation of GnRH neurons

The different antibodies and reagents used in the immunocytochemical studies listed below are summarized in Table 2.

**Table 2.** An overview of the reagents used for immunohistochemistry

	Immunofluorescent histochemistry (IF)	Immunoelectron microscopy (IEM)	Retrograde tract tracing
Antigens	Double-label IF VAChT/GFP (GnRH) YFP (ChR2-ChAT)/ChAT or Citrine (hM3Dq-ChAT)/ChAT Triple-label IF GnRH/VAChT/VGAT	Double-label IEM GFP (GnRH)/VAChT	Triple-label detection mCherry (AV)/GFP (Rabies)/Chat
Primary antibodies	Double-label IF Rabbit a-VAChT (1:5,000, RRID: AB_2571850, gift from M. Watanabe, Hokkaido University, Japan) Goat a-GFP (1:5,000, #AB5450, RRID: AB_304897, Abcam) or Rabbit a-GFP (1:1,000, #AB10145, RRID: AB_1587096, Millipore/Merck) Goat a-ChAT (1:500, #AB144P, RRID: AB_2079751, Merck/Millipore/Invitrogen) Triple-label IF Guinea pig a-GnRH (1:10,000, gift from E. Hrabovszky, IEM, Hungary) Rabbit a-VAChT (1:5,000) Goat a-VGAT (1:2,500, gift from M. Watanabe, Hokkaido University, Japan)	Double-label IF Goat a-GFP (1:2,500) Rabbit a-VAChT (1:10,000)	Triple-label detection Rabbit a-DS Red (1:2,000, #632496,Takara Bio/Clontech) Chicken a-GFP (1:1,000, #A10262, RRID: AB_2534023, Thermo Fisher Scientific) Goat a-ChAT (1:500)
Secondary antibodies used	Double-label IF Cy5-Dk a-Rb IgG (1:500–1,000, #711-175-152, RRID: AB_2340607, Jackson ImmunoResearch) Cy3-Dk a-Gt IgG (1:500–2,000, #705-165-147, RRID: AB_2307351, Jackson ImmunoResearch) Triple-label IF Cy5-Dk a-GP IgG (1:1,000, #706-175-148, RRID: AB_2340462, Jackson ImmunoResearch) A488-Dk a-Rb IgG (1:1,000, A-11055, RRID: AB_2534102, Molecular Probes) Cy3-Dk a-Gt IgG (1:2,000, #711-165-152, RRID: AB_2307443, Jackson ImmunoResearch)	Double-label IF 0.8 nm Gold-Dk a-Sh IgG (1:100, Electron Microscopy Sciences) Biot-Dk a-Rb IgG (1:500, #711-065-152, RRID: AB_2340593 Jackson ImmunoResearch)	Triple-label detection Cy3-Dk a-Rb IgG (1:2,000, #711-165-152, Jackson ImmunoResearch) FITC-Dk a-chicken IgG (1:500, #703-095-155, RRID: AB_2340356, Jackson ImmunoResearch) Cy5-Dk a-Goat IgG 1:1,000, #705-175-147, RRID: AB_2340415, Jackson ImmunoResearch)
Developing electron dense precipitate		ABC/Ni-DAB (1:500, PK-6100, VECTASTAIN ABC Kit, Standard, Elite)	

Dk, donkey; GP, guinea pig; Rb, rabbit; Gt, goat; Sh, sheep; ABC, avidin-biotin complex; Ni-DAB, nickel-enhanced diaminobenzidine.

#### Networking of cholinergic axons with GnRH neurons revealed by 3DISCO technique

Adult male GnRH-GFP transgenic mice (*n* = 6) were perfused transcardially with 4% paraformaldehyde (PFA, pH 7.6 in PBS) for 10 min at a flow rate of 4 ml/min. Brains were removed rapidly from the skulls and postfixed in the same fixative overnight at 4°C. Brains placed in a mouse brain mold were sliced manually at 1 mm thickness in the sagittal plane. Thereafter, double fluorescent immunocytochemistry (ICC) combined with the 3DISCO technique (Ertürk and Şengül, 2012) was applied with modifications (Vastagh et al., 2021). Briefly, the slices were rinsed several times in PBS, then permeabilized with 0.5% Triton X-100 in PBS containing 0.1% gelatin and 0.05% merthiolate (PBS-GT) for 3 d at room temperature (RT) under constant agitation. For fluorescent immunocytochemical double labeling, brain slices were incubated with a cocktail of goat, anti-GFP, and rabbit anti-VAChT primary antibodies for 7 d at RT. Several washes (6 × 60 min) in PBS-GT were followed by the incubation with a cocktail of Cy3-conjugated donkey anti-goat IgG and Cy5-conjugated donkey anti-rabbit IgG (1:500) secondary antibodies for 7 d at RT. Slices were then rinsed in PBS-GT for 6 × 60 min and then in PBS for 30 min. For dehydration and lipid removal steps, a series of increasing tetrahydrofuran (THF, Sigma-Aldrich) concentrations were used (50% THF overnight at 4°C, 80%, and 3 × 100% THF for 30 min at RT). For optical clearing, slices were immersed in dibenzyl ether (DBE, Sigma-Aldrich) for at least 15 min and then kept either in DBE for storage or covered using a glass slide with custom-made plastic supporting frames adjusted for the actual specimen thickness. The specimen was coverslipped with DBE for the later confocal microscopic analysis.

#### Quantitative 3D image analysis of cholinergic innervation of GnRH neurons

**Figure 2.**
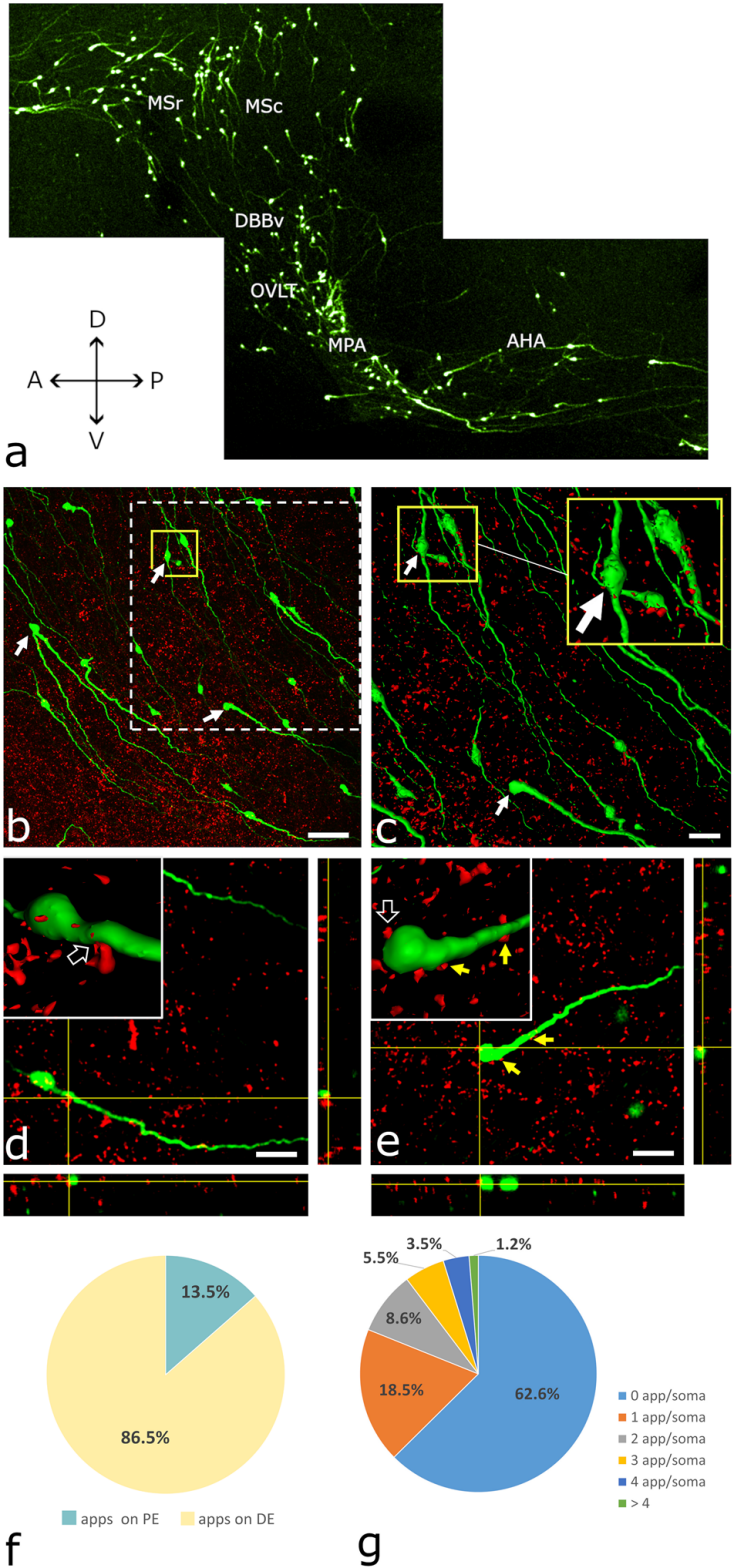
GnRH neurons receive cholinergic inputs as revealed by double immunostaining and the 3DISCO method. ***a***, Distribution of GnRH-GFP-IR elements revealed by 3DISCO. A thick brain slice (1 mm) cut in the paramedian sagittal plane. It represents the scanning areas used for image acquisition via confocal microscopy. ***b***, A stack of confocal image planes projected in the *z* direction shows many GnRH neurons (green channel, arrows) and VAChT-IR axon varicosities (red channel). ***c***, A part of the image stack shown by the dashed square in ***b*** was reconstructed as a 3D surface model using the ImageJ/Fiji software. Somata of GnRH neurons are clearly distinguishable (arrows). A representative reconstruction of two GnRH neurons captured from ***c*** is enlarged in the inset (yellow frame). Note the overlap of the two immunolabeled systems. ***d***, Axo-dendritic contact between a VAChT-IR axon and a GnRH neuron is shown in both the 3D surface model (inset, arrow) and orthogonal views of stacks (yellow crosshairs). ***e***, The inset shows an axo-somatic juxtaposition (empty arrow) in a 3D image. The same communication site is confirmed in orthogonal views (yellow crosshairs). Two other cholinergic axon varicosities (yellow arrows) juxtaposed to the same GnRH neuron indicate the existence of multiple contact sites. ***f***, The cholinergic axons communicate with GnRH dendrites (86.5%); the perikarya are less often targeted (13.5%). Apps on PE, appositions on perikarya; Apps on DE, appositions on dendrites. ***g***, About one-third (37.4%) of the analyzed GnRH perikarya received VAChT-IR inputs. The pie diagram depicts the percentage distribution of VAChT-IR axon beads juxtaposed to the same GnRH perikaryon. App/soma: apposition per soma. Scale bars: ***a***, 50 µm; ***b***, 25 µm; ***c*** and ***d***, 20 µm. MSr, medial septum, rostral part; MSc, medial septum, caudal part; DBBv, diagonal band of Broca, vertical part; OVLT, organum vasculosum of the lamina terminalis; MPA, medial preoptic area; and AHA, anterior hypothalamic area.

For quantitative evaluation of the interacting cholinergic and GnRH neuronal systems, stacks of images were acquired with a Nikon C2 laser scanning confocal system (Nikon Instruments Europe BV). To maximize the emitted signal intensity, we set sequential scanning mode in the software (NIS-Elements AR, Nikon). For Cy3-coupled labeling, an argon laser (561 nm) and a 595/40 nm emission filter (Nikon) were used. At the same time, to detect Cy5 emission, a He–Ne laser (637 nm) and a DAPI/Cy5 (custom 239415; Semrock) dual emission filters were applied. The sampling of interacting neuronal profiles was carried out in two slices per mouse brain and four adjacent frames of each sagittal brain slice. A total of 48 imaging areas were studied in six brains that contained the residence of GnRH neurons (Fig. 2).

For quantitative analysis, 20× objective magnification (Plan-Apo VC DIC N2; NA, 0.75; WD, 1 mm) was used at 1,024 × 1,024-pixel *x*–*y* image resolution. During the scanning process, the pinhole diameter was set to 1 AU (Airy unit) in the acquisition software. The optical thickness was 2 µm. The optical sectioning was adjusted to match pinhole size (*z*-step interval, 1.2 µm).

All GFP-IR perikarya were counted in the *z*-stacks of scanned areas. In the orthogonal view of the *z*-stacks, GFP-IR neurons that received VAChT-IR axonal appositions were also counted. The ratio of GFP-IR perikarya targeted by VAChT axons was calculated. The ratio of all GFP-IR dendrites to those contacted by VAChT-IR axons was determined similarly.

#### Detecting VGAT in the cholinergic afferents of GnRH neurons by triple-label fluorescent ICC

Adult, transgenic mice (*n* = 4) were perfused transcardially with 4% PFA as described above. Brains were removed rapidly from the skulls and postfixed in the same fixative overnight at 4°C. On the next day, brains were sectioned in the coronal plane at 25 µm thickness using a Leica VT 1000S Vibratome (Leica Microsystems). Sections containing the medial septum (MS), diagonal band of Broca (DBB), and mPOA were pretreated with 0.5% H_2_O_2_ and permeabilized with 0.5% Triton X-100 for 20 min. Sections were incubated first in a cocktail of guinea pig anti-GnRH, rabbit anti-VAChT, and goat anti-VGAT primary antisera (48 h, at 4°C) and after thorough rinsing in PBS in a cocktail of the corresponding secondary antibodies, that is, Alexa Fluor 488-conjugated donkey anti-rabbit IgG; Cy3-conjugated donkey anti-goat IgG; and Cy5-conjugated donkey anti-guinea pig IgG. All sera were diluted in PBS containing 2% normal horse serum.

#### Immunoelectron microscopy

GnRH-GFP transgenic mice (*n* = 10) were anesthetized and perfused transcardially with 10 ml of 0.01 M PBS, pH 7.4; followed sequentially by 30 ml of fixative containing 4% acrolein and 2% PFA in PBS, pH 7.4; and then by 40 ml of 4% PFA in PBS, pH 7.4. The brains were removed and stored in 4% PFA in 0.1 M PBS overnight at 4°C. Serial 25-µm-thick coronal sections were cut on a vibratome through the MS-DBB and the mPOA. The sections were treated with 1% sodium borohydride in 0.1 M PBS for 30 min, followed by 0.5% H_2_O_2_ in PBS for 15 min, cryoprotected in 15% sucrose in PBS for 15 min at RT, and in 30% sucrose in PBS overnight at 4°C. Thereafter, they were incubated with 2% normal horse serum for 20 min to block the nonspecific binding of antibodies. The tissue penetration of antibodies was facilitated with Photo-Flo (Kodak Photo-Flo 200 Solution) added to the used antibodies at a concentration of 0.3%.

The pretreated sections were placed in a mixture of goat anti-GFP serum (1:2,500) and rabbit anti-VAChT serum (1:10,000) for 4 d at 4°C. After rinsing in PBS and 0.1% cold water fish gelatin and 1% BSA in PBS, the sections were incubated in mixture of biotinylated donkey anti-rabbit IgG and 0.8 nm colloidal gold-conjugated donkey anti-sheep IgG (Electron Microscopy Sciences) diluted at 1:100 and biotinylated anti-rabbit IgG diluted at 1:500 in PBS containing 0.1% cold water fish gelatin and 1% BSA overnight at 4°C. After washing in PBS, the sections were fixed in 1.25% glutaraldehyde in 0.1 M PBS for 10 min at RT. After further rinsing in PBS, the sections were washed in Aurion ECS buffer (Aurion; 1:10, diluted in distilled water). The sections were further rinsed in 0.2 M sodium citrate, pH 7.5, and then the gold particles were silver intensified with the Aurion R-Gent SE-LM Kit. The sections were placed in 0.05% gold chloride for 2 × 5 min at RT and washed in 0.2 M sodium citrate, pH 7.5, and in 3% sodium thiosulfate solution for 10 min each at RT. Then the sections were treated in avidin/biotin-peroxidase complex (Vectastain Elite ABC Elite 1:500, Vector Laboratories) and the VAChT-IR was developed in 0.05% Ni-DAB /0.005% H_2_O_2_ in 0.05 M Tris buffer, pH 7.6.

The sections were processed for electron microscopy, first osmicated for 30 min (OsO_4,_ 1%) at RT and then treated with 2% uranyl acetate in 70% ethanol for 30 min. Following dehydration in an ascending series of ethanol and propylene oxide (Sigma), the sections were flat embedded in Araldite 6005 epoxy resin (EMS) on liquid release agent-coated slides and polymerized at 56°C for 2 d. After polymerization, 60–70 nm-thick ultrathin sections were cut with a Leica UCT ultramicrotome (Leica Microsystems). The ultrathin sections were mounted onto Formvar-coated, single-slot grids and examined with a Hitachi H-7100 transmission electron microscope (Hitachi).

#### Cre-dependent retrograde rabies virus tract tracing

##### Stereotaxic surgery

A cocktail of helper viruses (pAAV-EF1a-FLEX-TVA-mCherry/pAAV-CAG-FLEX-oG-WPRE-SV40pA in a ratio of 1:1; Salk Institute) was delivered by a single injection into the MS/DBB region [Bregma anteroposterior (AP), +0.85 mm; dorsoventral (DV), −4.5 mm; and mediolateral (ML), 0 mm] and bilateral injections into the MPA (Bregma AP, +0.49 mm; DV, −5 mm; and ML, ±0.25 mm) of male GnRH-Cre mice (*n* = 15). Three weeks later, bilateral injections were made with the rabies ΔG-EnvA-eGFP virus (BRVenvA-2C, Charité–Universitätsmedizin Berlin, Viral Core Facility) into the helper virus-infiltrated territory (Bregma AP, +0.6; DV, −5 mm; and ML, ±0.25 mm). Due to the expression of the transgenes, helper virus-infected cells became red (in a Cre-dependent manner), while rabies virus-infected cells became green (at the injection site, in a random manner). The starter cells expressing both transgenes became yellow. The helper virus-infected cells contained an upgraded version of the rabies glycoprotein (oG) that has increased the *trans*-synaptic labeling potential (Kim et al., 2016). After 8–14 d of survival, mice were perfused with 4% PFA, brains were removed, and 30 µm-thick sections were cut on a freezing microtome (Reichert).

##### Immunohistochemical identification of rabies-infected cholinergic neurons

The fluorescent marker proteins mCherry and GFP expressed in response to the viral infections and the cholinergic marker enzyme ChAT were detected by a triple-label immunofluorescent technique using rabbit anti-DS Red IgG, chicken anti-GFP, and goat anti-ChAT primary antibodies and Cy3-, FITC-, and Cy5-conjugated donkey anti-rabbit, anti-chicken, and anti-goat secondary antibodies, respectively. Sections were mounted, coverslipped by ProLong Gold Antifade, and scanned by a slide scanner (Pannoramic Scan; 3DHISTECH) using 10× objective magnification. Selected sections underwent confocal analyses using a Nikon A1 confocal microscope at 20× and 60× magnification.

##### Immunohistochemical controls

The specificity of the primary antisera used has been reported previously. Accordingly, increasing dilutions of the primary antibodies resulted in a gradual decrease and eventual disappearance of the immunostaining; omission of the primary antibodies or their preabsorption with corresponding peptide antigens resulted in a complete loss of the immunostaining. The secondary antibodies used here were designed for multiple labeling and were preabsorbed by the manufacturer with IGs from several species, including the one in which the other primary antibody had been raised.

### Real-time polymerase chain reaction (RT-PCR)

#### Cytoplasm sampling

GnRH cytoplasmic samples were harvested from acute forebrain slices of adult male mice containing GnRH-GFP neurons using a patch pipette. A total of seven samples were processed, with 10 cytoplasmic fractions per sample. The tips of the glass pipettes were broken into PCR tubes, and then cDNA was synthesized from the sample RNA using SuperScript IV VILO Master Mix (#11766050, Thermo Fisher Scientific) according to the manufacturer’s instructions.

#### PCR

Preamplification ran for 14 cycles (TaqMan PreAmp Master Mix Kit, Thermo Fisher Scientific). Gene expression analyses were performed on a ViiA 7 RT-PCR system (Thermo Fisher Scientific) using TaqMan assays (Thermo Fisher Scientific) according to the manufacturer’s instructions for the following target genes: *Gapdh*, *Hprt*, *GnRH1*, *Chrm1*, *Chrm2*, *Chrm3*, *Chrm4*, *Chrm5*, *Chrna2*, *Chrna3*, *Chrna4*, *Chrna5*, *Chrna6*, *Chrna7*, *Chrnb1*, *Chrnb2*, *Chrnb3*, *Chrnb4*, *Chrnd*, *Chrne*, and *Chrng*. The RT-PCR experiments were programmed to run for 40 cycles. Expression of the *Gnrh* gene was detectable in all seven cell pools. The mean expression of *Gapdh* and *Hprt* genes in each of the cytoplasmic sample pools (CPs) was used as a reference (Ct_ref_). Gene expression level (G) of the target genes was calculated as follows: G = 2^−ΔCt,^ where ΔCt is the difference in threshold cycle between the target and reference genes (ΔCt = Ct_gene_–Ct_ref_; Riedel et al., 2014).

### Electrophysiology

#### Slice electrophysiology

Brain slice preparation was carried out as described earlier (Farkas et al., 2010). Briefly, after decapitation, the heads were immersed in ice-cold, low-Na cutting solution and continuously bubbled with carbogen, a mixture of 95% O_2_ and 5% CO_2_, and the brains were removed rapidly from the skull. The cutting solution contained the following (in mM): 205 sucrose, 2.5 KCl, 26 NaHCO_3_, 5 MgCl_2_, 1.25 NaH_2_PO_4_, 1 CaCl_2_, and 10 glucose. Hypothalamic blocks were dissected, and 220-µm-thick coronal slices were prepared from the mPOA with a VT 1000S Vibratome (Leica Microsystems) in the ice-cold, low-Na, oxygenated cutting solution. The slices containing the POA were transferred into artificial cerebrospinal fluid (aCSF; in mM): 130 NaCl, 3.5 KCl, 26 NaHCO_3_, 1.2 MgSO_4_, 1.25 NaH_2_PO_4_, 2.5 CaCl_2_, and 10 glucose, bubbled with carbogen, and left for 1 h to equilibrate. Equilibration started at 33°C, and it was allowed to cool down to RT.

Recordings were carried out in carbogenated aCSF at 33°C. Axopatch 200B patch-clamp amplifier, Digidata 1322A data acquisition system, and pClamp 10.4 software (Molecular Devices) were used for recording. Neurons were visualized with a BX51WI IR-DIC microscope (Olympus). The patch electrodes (OD, 1.5 mm; thin wall; World Precision Instruments) were pulled with a Flaming/Brown P-97 puller (Sutter Instrument).

GnRH-GFP neurons were identified by brief illumination at 470 nm using an epifluorescent filter set based on their green fluorescence, typical fusiform shape, and characteristic topography (Suter et al., 2000b).

#### Whole-cell patch-clamp recording

Whole-cell patch-clamp measurements started with a control recording (1–5 min), then the selected receptor ligand was pipetted directly into the aCSF-filled measurement chamber containing the brain slice in a single bolus, and the recording continued for a further 7–10 min. Pretreatment with the extracellularly applied antagonists started 10 min before starting the recording, and the antagonist was continuously present in the aCSF during the electrophysiological recording. Each neuron served as its own control when drug effects were evaluated.

The membrane current and the mPSCs in GnRH neurons were measured as described earlier (Farkas et al., 2010). Briefly, the neurons were voltage clamped at −70 mV of holding potential. The intracellular pipette solution contained the following (in mM): 10 HEPES, 140 KCl, 5 EGTA, 0.1 CaCl_2_, 4 Mg-ATP, and 0.4 Na-GTP, pH 7.3 with NaOH. The resistance of the patch electrodes was 2–3 MΩ. Only cells with a low holding current (10 pA) and a stable baseline were used. Input resistance (*R*_in_), series resistance (*R_s_*), and membrane capacitance (*C_m_*) were also measured before and after each treatment by using 5 mV hyperpolarizing pulses. To ensure consistent recording qualities, only cells with *R_s_* < 20 MΩ, *R*_in_ > 500 MΩ, and *C_m_* > 10 pF were accepted. Spike-mediated transmitter release was blocked in all mPSC experiments by adding the voltage-sensitive Na channel inhibitor tetrodotoxin (TTX; 660 nM, Tocris) to the aCSF 10 min before mPSCs were recorded. Time distribution graphs of frequencies were generated using 30 s time bins, shifted by 5 s steps, to show the time courses of the effect of substances.

Action potentials (APs) were recorded in whole-cell current-clamp mode at 0 pA. After a control period (1–3 min), the agonists were applied, and the recording continued for 8–10 min. The antagonist pretreatments started 10 min before starting the recording. Each neuron served as its own control when drug effects were evaluated.

To block GABA_A_-R and glutamate-R–mediated synaptic inputs to GnRH neurons, the slices were pretreated extracellularly with picrotoxin (100 µM, Tocris) and kynurenic acid (2 mM, Sigma) 10 min before the recording started.

The intracellularly applied tetrahydrolipstatin (THL, Tocris, 10 µM) was added to the intracellular (pipette) solution. After achieving the whole-cell configuration, measurements started after 15 min of equilibration to reach a stable intracellular milieu.

#### Optogenetics

The applied technique has recently been published (Vastagh et al., 2021) with a slight modification. A LED light source (CoolLED pE-100, 470 nm) was fitted to the microscope, illuminating the sample via the objective lens. Whole-cell patch-clamp measurements were carried out to record mPSCs in voltage-clamp or APs in current-clamp mode in GnRH neurons in acute brain slices of triple-transgenic Chat-Cre-ChR2-GnRH-GFP adult male mice. The neurons were voltage clamped at a pipette holding potential −70 mV (for mPSCs) and current clamped at 0 pA (for APs). The duration of a LED pulse was 5 ms.

For evoked mPSCs (emPSCs), LED pulses were applied at 0.2 Hz or 5 Hz (60 runs total; each run was 5 s long), and then records of the emPSC responses of the 60 runs were averaged (9 neurons from 5 mice). The AChR receptor antagonists, mecamylamine and atropine, or the GABA_A_-R blocker picrotoxin was added to the aCSF, and 10 min later, the measurement was repeated.

Measurement of the firing rate or mPSC frequency changes was carried out using LED pulses. The measurements started with a recording with no LED (for control purposes, 30 s for APs, 2 min for mPSCs) and with a subsequent LED train of 5 Hz illumination period (60 s for APs, 2 min for mPSCs). The AChR antagonists mecamylamine and atropine were added to the aCSF, and 10 min later, the measurement was carried out in the presence of the antagonists.

## Results

### Pharmacogenetic activation of the central cholinergic system in vivo increases secretion of LH in orchidectomized mice

In the Chat-G_q_ (hM3Dq) DREADD mouse line, the expression of DREADD receptor was observed exclusively in ChAT-IR, cholinergic neurons (Fig. 1*a*) indicating the specificity of the pharmacogenetic targeting. The serum LH levels evaluated by ELISA technique demonstrated a surge-like release profile in the CNO-treated group, which was not observed in the vehicle group (Fig. 1*b*). There was a significant difference in the post-treatment mean LH levels (*p* = 0.006; ANCOVA; *F*_(2,7)_ = 11.38). The contrast estimate for the difference between the vehicle and CNO groups was 13.37 ± 5.37 ng/ml (mean ± SEM), and the CNO-treated group had a significantly higher mean LH level compared with vehicle (*p* = 0.042; Tukey’s post hoc; *t* = 2.49; Cohen’s *d* = 0.57). The adjusted mean LH levels of the post-treatment period were 8.10 ng/ml (SEM = 3.26; 95% CI [0.40, 13.77]) in the vehicle and 21.46 ng/ml (SEM = 3.26; 95% CI [15.80, 29.16]) in the CNO group (Fig. 1*c*).

Similarly, significant difference was measured in the post-treatment basal LH levels (*p* = 0.001437; ANCOVA; *F*_(2,7)_ = 19.21). The contrast estimate for the difference between the vehicle and CNO groups was 10.02 ± 3.35 ng/ml (mean ± SEM): CNO-treated group had a significantly higher mean LH level compared with vehicle (*p* = 0.0203; Tukey’s post hoc; *t* = 2.99; Cohen’s *d* = 0.64). The adjusted basal LH means of the post-treatment period were 6.84 ng/ml (SEM = 2.03; 95% CI [2.04, 11.6]) in the vehicle and 16.9 ng/ml (SEM = 2.03; 95% CI [12.10, 21.7]) in the CNO group, respectively (Fig. 1*d*).

LH AUC as a function sampling period (60 min) was significantly different in the post-treatment period (*p* = 0.0072; ANCOVA; *F*_(2,7)_ = 10.81). The contrast estimate for the LH AUC difference between the vehicle and CNO groups was 815.5 ± 308.7 ng/ml * 60 min (mean ± SEM): the CNO-treated group had a significantly higher LH AUC compared with vehicle (*p* = 0.0333; Tukey’s post hoc; *t* = 2.64; Cohen’s *d* = 0.59). The adjusted LH AUC means of the post-treatment period were 444 ng/ml * 60 min (SEM = 189; 95% CI [−3.00, 891]) in the vehicle and 1,269 ng/ml * 60 min (SEM = 189; 95% CI [812, 1,706]) in the CNO group (Fig. 1*e*).

LH amplitude analysis showed that there was no significant difference between the groups (*p* = 0.061; ANCOVA; *F*_(2,7)_ = 4.283). The adjusted LH amplitude means of the post-treatment period were 3.76 ± 1.91 ng/ml in the vehicle and 7.36 ± 1.91 ng/ml in the CNO group (Fig. 1*f*). The difference in the LH peak frequencies (Fig. 1*g*) did not reach statistical significance, either (*p* = 0.2378; ANCOVA; *F*_(2,7)_ = 1.776). The adjusted LH pulse frequency means of the post-treatment period were 3.58 ± 0.47/60 min in the vehicle and 4.82 ± 0.47/60 min in the CNO group.

### Networking of central cholinergic neurons with GnRH neurons

#### GnRH neurons receive cholinergic innervation

For structural evaluation and quantitative analysis of the putative interneuronal communication of the central cholinergic and GnRH systems, double immunofluorescence labeling combined with the 3DISCO method was used on paramedian sagittal slices of the GnRH-GFP transgenic mouse brain that contained the distribution sites of GnRH neurons in the MS, the vertical limb of the DBB, the region of the organum vasculosum laminae terminalis (OVLT), the mPOA, and the anterior hypothalamic area (Fig. 2*a*). The cholinergic axons were identified by their VAChT content, whereas GnRH-IR profiles were visualized by immunostaining of the expressed GFP. GnRH neurons were found to overlap with VAChT-IR axons (Fig. 2*b*,*c*) distributed in the sampled regions. Altogether, 4,899 optical slices (mean ± SEM: 816 ± 64 per brain) were evaluated. In each optical slice, the number of VAChT-IR axons juxtaposed to GnRH-IR perikarya and dendrites was determined. Juxtapositions were justified with high precision in the orthogonal view layout. Many VAChT-IR axon varicosities (1,040 ± 96 SEM; *n* = 6) were identified in juxtaposition with GnRH-IR profiles (Fig. 2*d*,*e*). Most of them (86.5 ± 3.2%) targeted GnRH dendrites (Fig. 2*d*) and only a smaller fraction (13.5 ± 3.2%) contacted GnRH perikarya (909 ± 100.12 dendritic vs 131 ± 23.68 somatic input; *n* = 6; paired *t*-test; *p* = 0.0008; two-tailed; Fig. 2*e*,*f*). In these samples, as an average, 37.4 ± 5.1% of reconstructed GnRH-IR perikarya (186 ± 20) received cholinergic inputs (68 ± 9.66 innervated vs 118 ± 16.9 noninnervated GnRH-IR perikarya; *n* = 6; paired *t*-test; *p* = 0.0454; two-tailed; Fig. 2*g*).

#### Ultrastructural correlates of networking

**Figure 3.**
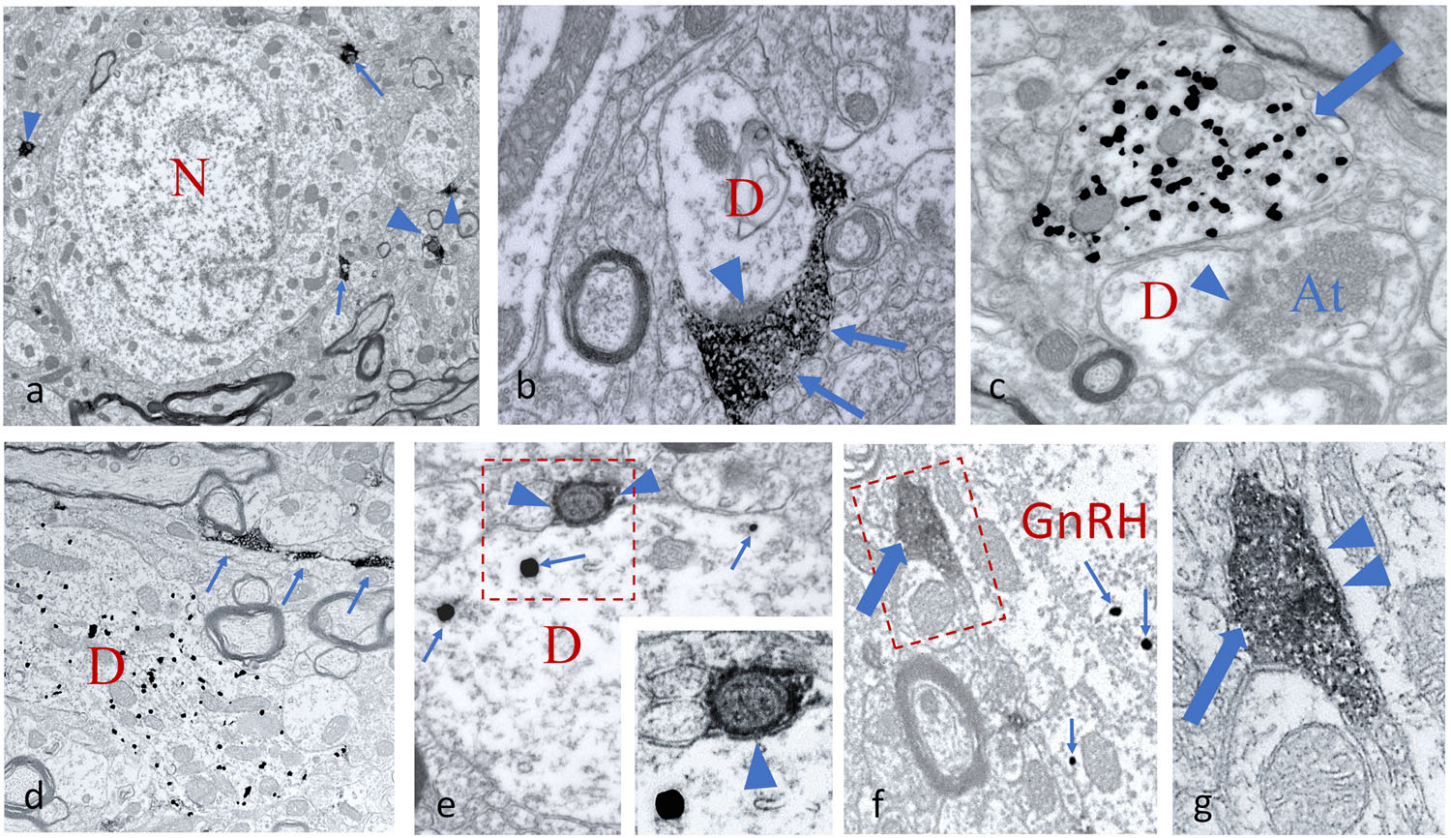
Ultrastructural detection of cholinergic axons and GnRH-IR profiles in the mPOA. ***a***, VAChT-containing axons labeled by Ni-DAB chromogen are distributed in the neuropil of the mPOA, contacting cell bodies (arrows) and dendrites (arrowheads). A nonlabeled neuron (N) is in the center of micrograph. ***b***, A Ni-DAB chromogen-labeled cholinergic axon (arrows) synapses (arrowhead) on a cross-sectioned dendrite (D). ***c***, Appearance of a cross-sectioned GnRH dendrite (arrow) filled with silver-intensified, colloidal gold particles. A nearby unlabeled dendrite (D) receives a synapsing (arrowhead) axon terminal (At). ***d***, Simultaneous detection of a longitudinally sectioned GnRH dendrite (D) identified by silver-coated colloidal gold particles and a varicose, longitudinally cut VAChT-IR axon (arrows) labeled by Ni-DAB chromogen. ***e***, Cross-sectioned GnRH-IR dendrite (D) communicating with a small caliber cholinergic axon (arrowheads). Arrows point to the metallic label within the GnRH profile. The enframed region is shown at higher power in the inset, and the arrowhead points to the postsynaptic membrane. ***f***, Detail from a GnRH perikaryon (GnRH) that receives a cholinergic axon (thick arrow). Intensified, colloidal gold particles are labeled by small arrows. The communication site is enframed. ***g***, High-power view of the enframed area in ***f***. The labeled, cholinergic axon (thick arrow) filled with small-sized vesicles establishes a synapse (arrowheads) with the GnRH perikaryon. Scale bars: ***a***, 2 µm; ***d***, 1 µm; ***b***, ***c***, 500 nm; ***e–g***, 250 nm.

The networking among VAChT-IR axons and GnRH neurons was also confirmed at the ultrastructural level using pre-embedding double ICC. For the labeling of VAChT-IR axons, nickel-3,3 diaminobenzidine (Ni-DAB) chromogen was used (Fig. 3*a*,*b*), while the GnRH-IR profiles were visualized by silver-intensified colloidal gold particles (Fig. 3*c*,*d*). In the residence area of hypophysiotropic GnRH neurons, the MS-DBB-OVLT continuum, VAChT-expressing axons appeared in juxtaposition to cell bodies and dendrites (Fig. 3*a*) and formed synapses (Fig. 3*b***)**. Based upon the different physicochemical properties of the used chromogens, the GnRH and VAChT-IR profiles were clearly distinguishable from each other even at medium-power electron microscopic screening (Fig. 3*d*). The juxtaposition of cholinergic axon beads to dendritic and somatic domains of GnRH neurons without glial process interposition was confirmed at the ultrastructural level. Tracing the juxtaposed profiles through a series of ultrathin sections occasionally revealed the synaptic engagement of VAChT-IR, cholinergic axons with dendrites (Fig. 3*e*), and perikarya (Fig. 3*f*,*g*) of GnRH neurons, suggesting a direct regulatory influence of the cholinergic system upon the GnRH neuron assembly.

#### Identification of cholinergic neuronal source supplying innervation of GnRH neurons

**Figure 4.**
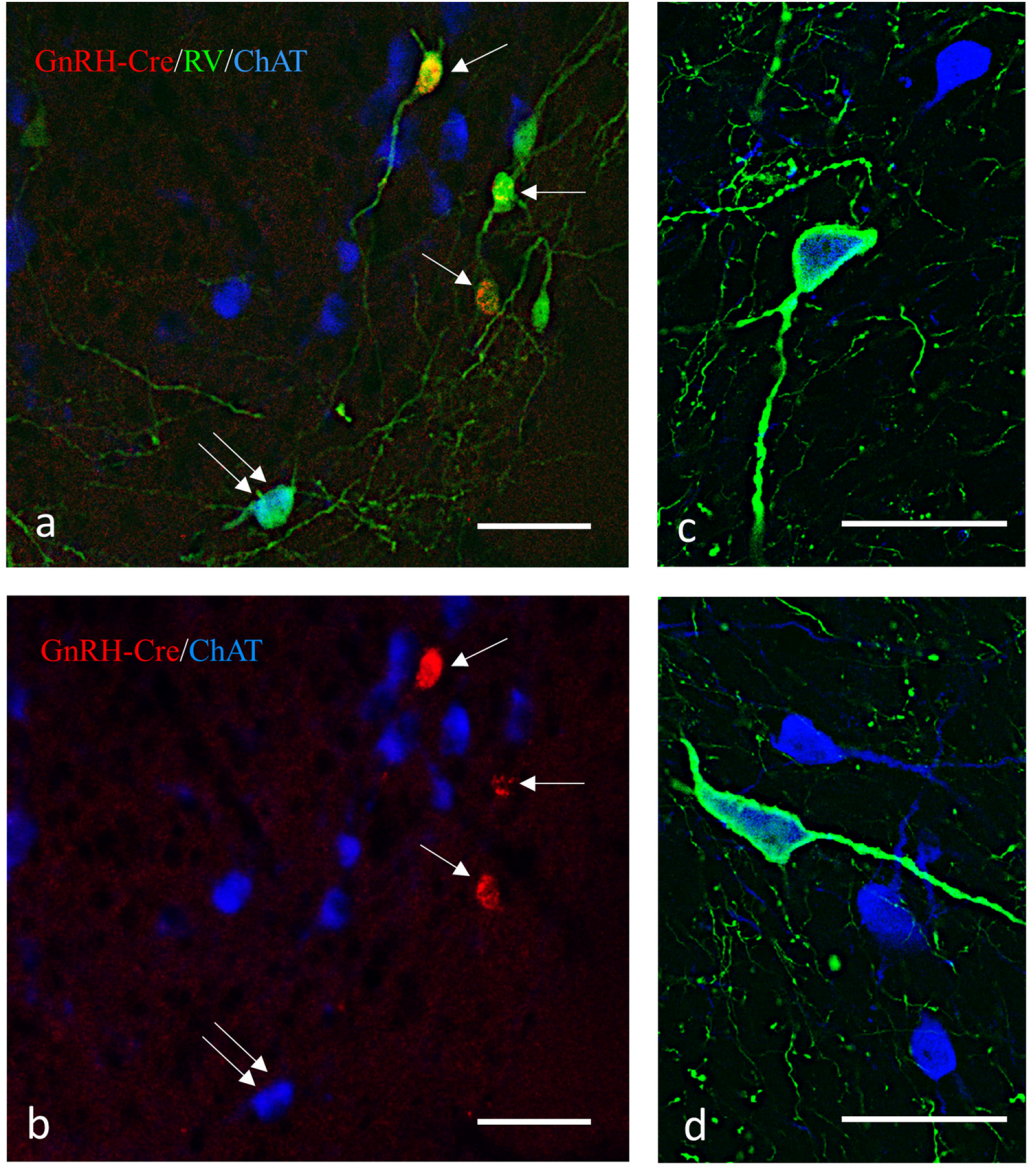
Viral retrograde tracing of cholinergic afferents of GnRH neurons. Monosynaptic tracing of primary afferents to GnRH-Cre neurons and subsequent immunohistochemical processing of brain sections identify AAV-infected, Cre-positive neurons expressing mutated TVA and G-protein fused to mCherry (red), rabies virus-GFP infected cells (green), and Chat-IR cells (blue). Contrary to the mCherry signal, the green fluorescence fills both the perikarya and the processes. ***a***, Starter cells (single arrows) express both mCherry and GFP, whereas the primary afferent neurons produce only GFP. Double arrows point to a cholinergic primary afferent neuron (in turquoise) having a mixture of both green and blue signals. ***b***, Validation of double-labeled neurons shown in ***a*** by presenting the fluorescent signals in single channels. ***c***, ***d***, Examples of Chat-IR cells (blue) labeled with the rabies GFP signal (green) in the septo-diagonal band area of the mouse brain. Scale bar, 50 µm.

To figure out which of the brain regions host cholinergic cells innervating GnRH neurons, a mono-trans-synaptic retrograde tract tracing technique was used in combination with ICC. GnRH-Cre neurons (Fig. 4*a*,*b*) expressing TVA receptor and G-proteins became selectively infected by the modified, envelope protein A (EnvA)-coated, G-deleted rabies virus (RVdG). GnRH neurons expressed the TVA-GFP/TVA receptor protein as the precursor neurons for rabies infection (Fig. 4*a*). No mCherry fluorescence of helper virus origin was seen in wild-type mice, showing that the viral activation was Cre dependent. Rabies virus-labeled neurons forming the primary afferents of the GnRH-Cre cells appeared throughout the entire length of the neuroaxis, with the highest number observed in the vicinity of the starter cells. Proceeding from the POA toward the brainstem, their number gradually diminished. Detection of GFP fluorescence, an indicator of rabies virus transfection, in ChAT-IR neurons identified the source of cholinergic afferents to GnRH neurons. These double-labeled neurons were in the MS and the DBB (Fig. 4*c*,*d*). No primary cholinergic afferent neurons were identified in other parts of the forebrain or in the brainstem, indicating that the cholinergic regulatory input is local to the residence of GnRH neurons.

### Hypophysiotropic GnRH neurons express various nAChR and mAChR

**Figure 5.**
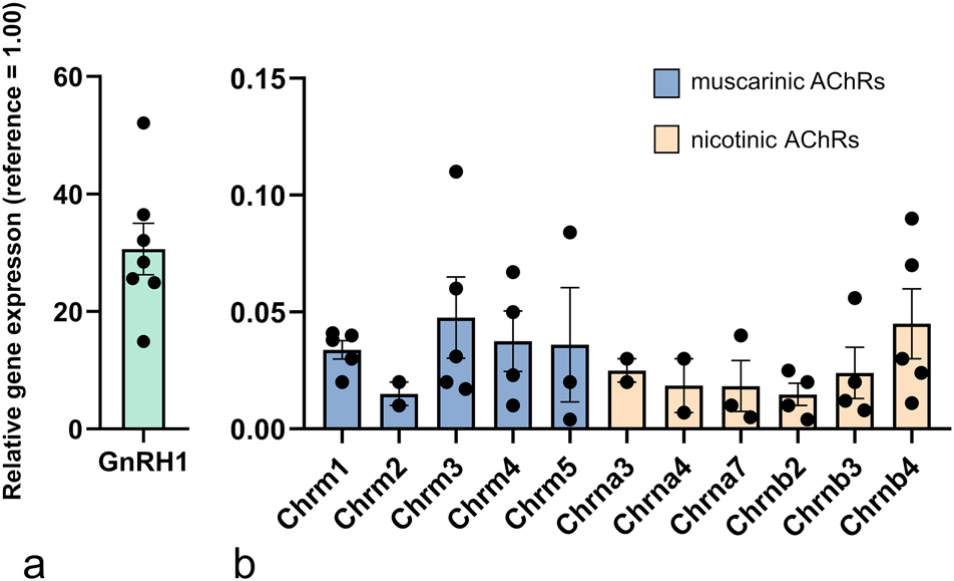
Expression of nAChR and mAChR genes in GnRH neurons. Relative expression of the Gnrh1 gene (***a***) and various nicotine and muscarine acetylcholine receptor genes (***b***) in CPs of GnRH neurons were revealed by qRT-PCR. The colored bars represent the mean relative gene expression values normalized to the mean of two housekeeping genes, with expression level set to 1, as reference. Primer probes used for the RT-PCR study are shown in Extended Data Figure 5-1.

Cytoplasmic samples (*n* = 70) were collected from GnRH-GFP neurons by patch pipette aspiration, sorted into separate CPs (*n* = 7), and examined by qRT-PCR. The primer probe sets used for detection of expression of genes encoding for mAChR and nAChR, housekeeping genes (*Hprt*, *Gapdh*), and *Gnrh1* are summarized in Extended Data Figure 5-1. Examination of the sample groups revealed the relative mRNA expression level of genes encoding for the following nicotinic receptor subunits in GnRH neurons (mean ± SE; Fig. 5*b*): α3 subunit (*Chrna*3) 0.026 ± 0.006 (detected in 2 of 7 CPs), α4 subunit (*Chrna4*) 0.016 ± 0.009 (2 of 7 CPs), α7 subunit (*Chrna7*) 0.018 ± 0.009 (3 of 7 CPs), β2 subunit (*Chrnb2*) 0.015 ± 0.005 (4 of 7 CPs); β3 subunit (*Chrnb3*) 0.023 ± 0.11 (4 of 7 CPs), and β4 subunit (*Chrnb4*) 0.047 ± 0.016 (5 of 7 CPs). Regarding muscarinic receptors (M1–M5), muscarinic type 1 (*Chrm1*) 0.034 ± 0.004 (5 of 7 CPs), type 2 (*Chrm2*) 0.011 ± 0.006 (2 of 7 CPs), type 3 (*Chrm3*) 0.048 ± 0.016 (5 of 7 CPs), type 4 (*Chrm4*) 0.038 ± 0.013 (4 of 7 CPs), and type 5 (*Chrm5*) 0.037 ± 0.024 (3 of 7 CPs) receptor genes were also measured in the samples (Fig. 5*b*). The expression of the *Gnrh* gene (30.2 ± 4.68) was confirmed in all samples (Fig. 5*a***)**.

10.1523/JNEUROSCI.1780-23.2024.f5-1 Figure 5-1 Primer probes used for the quantitative RT-PCR study. Download Figure 5-1, DOCX file.

### Cholinergic drugs change the electrophysiological activity of GnRH neurons in acute brain slice

#### Carbachol triggers ion current and modifies mPSCs of GnRH cells

**Figure 6.**
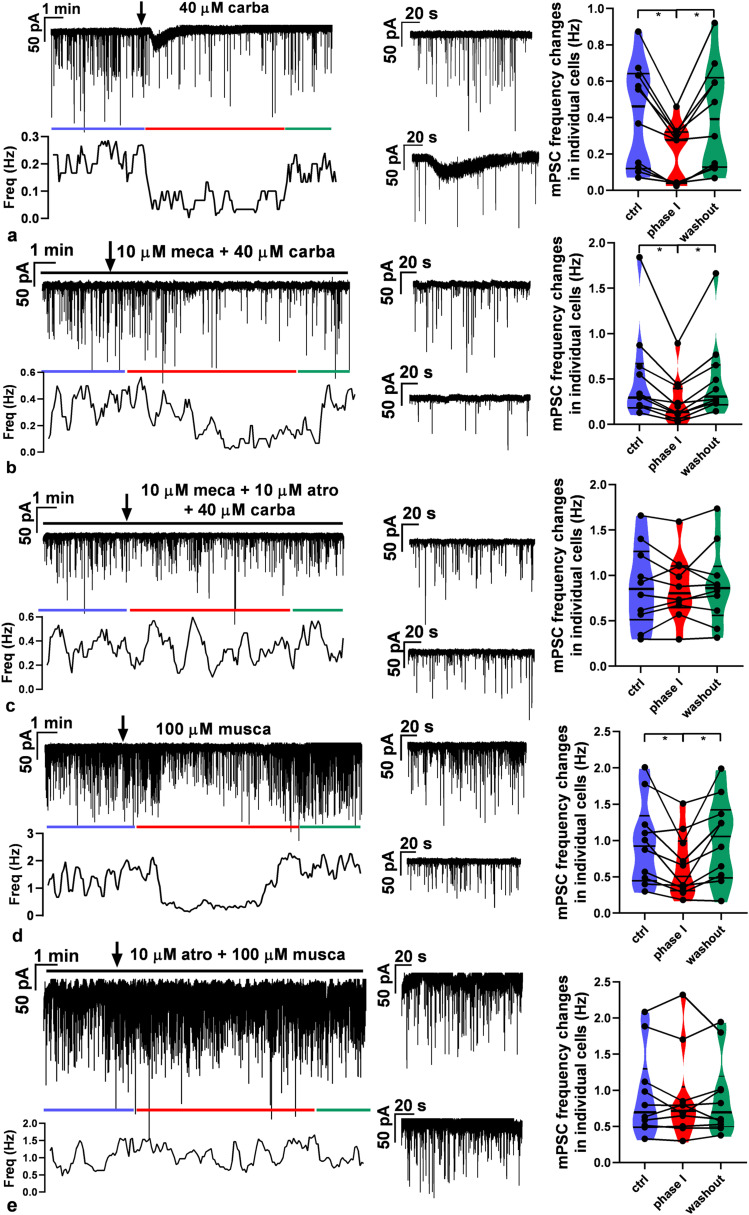
Effect of carbachol on the membrane current and mPSCs of GnRH neurons. Representative recordings illustrate that carbachol (40 µM) first evoked an inward current and then an inhibitory action on mPSCs of GnRH neurons in the presence of TTX (660 nM). ***a***, Carbachol triggered an inward current, followed by a slower inhibitory effect on the frequency of mPSCs. ***b***, Mecamylamine pretreatment (10 µM) revealed that the inward current is related to nicotinic receptors. ***c***, Simultaneous pretreatment with mecamylamine and atropine (10  µM) eliminated both the inward current and the inhibitory effect of carbachol. ***d***, Muscarine (100  µM) evoked exclusively the inhibitory effect upon mPSCs. ***e***, This inhibitory effect was totally abolished by atropine pretreatment. *p < 0.05. Horizontal black lines above the recordings show the presence of various AChR receptor subtype-specific inhibitors (mecamylamine, atropine) in the aCSF. The arrow shows the time of administration of carbachol or muscarine. Zoomed 2.5-min-long recordings of control (top graph) and treated (bottom graph) periods are besides the recordings. Changes in the frequency of mPSCs in individual GnRH neurons are shown besides these zoomed recordings. The colored lines under the recordings match the colors of the violin graphs and represent the various phases. The frequency distribution of the mPSCs is graphed under each recording. carba, carbachol; atro, atropine; musca, muscarine; and meca, mecamylamine. Details of the statistical analysis are provided in Extended Data Figure 6-1.

We studied the effects of activation of nAChR and mAChR by the AChR agonist, carbachol, on the membrane current and mPSCs in voltage-clamp mode at −70 mV. Application of carbachol in a single bolus triggered a transient inward current (amplitude, −36.9 ± 3.09 pA; Student’s *t*-test; *N/n* = 3/9; *p* = 0.0001; *t* = 11.95; df = 8; duration, 47.9 ± 12.34 s; Fig. 6*a*). Thereafter, the frequency of mPSCs significantly decreased which was then washed out (in Hz: control, 0.41 ± 0.091; phase I, 0.21 ± 0.050; wash out, 0.41 ± 0.093; Fig. 6*a*). We hypothesized that the inward current is caused by nAChR. Therefore, we next pretreated the brain slices with mecamylamine (nonselective nAChR antagonist, 10 µM), and then carbachol was added to the aCSF (Fig. 6*b*). The disappearance of the inward current confirmed that nAChR activation is involved in the effect of carbachol on membrane current. Nevertheless, the significant decrease in the frequency of the mPSCs was still observable (in Hz: control, 0.54 ± 0.16; phase I, 0.26 ± 0.083; wash out, 0.51 ± 0.14; Fig. 6*b*) raising the possibility that mAChRs play a role in the regulation of mPSC frequencies. Thus, next, the slices were pretreated with a cocktail of mecamylamine (10 µM) and the nonselective mAChR antagonist, atropine (10 M), and then carbachol was added to the aCSF (Fig. 6*c*). The inward current disappeared, and the frequency of mPSCs remained unchanged in this experiment (in Hz: control, 0.88 ± 0.14; phase I, 0.87 ± 0.11; wash out, 0.89 ± 0.14; Fig. 6*c*).

10.1523/JNEUROSCI.1780-23.2024.f6-1 Figure 6-1 Two-way ANOVA and Tukey's post-hoc tests of mPSC frequency data in Fig. 6. Download Figure 6-1, DOCX file.

To confirm the regulatory role of mAChRs in the decrease in the frequency of mPSCs, in the next experiment, the mAChR agonist muscarine (100 µM) was added to the aCSF in a single bolus. The measurements revealed that no inward current was evoked by muscarine. Nevertheless, a significant decrease in the frequency of mPSCs was detected which was then washed out (in Hz: control, 0.97 ± 0.18; phase I, 0.66 ± 0.14; wash out, 1.0 ± 0.18; Fig. 6*d*). However, atropine pretreatment abolished this change in frequency (in Hz: control, 0.94 ± 0.19; phase I, 0.91 ± 0.20; wash out, 0.91 ± 0.17; Fig. 6*e*).

Zoomed recordings from the control and the phase I periods are graphed next to each recording.

These results of the frequency changes are summarized in the graphs beside the recordings showing that mAChRs play a pivotal role in the decrease in frequency of mPSCs.

#### ACh and carbachol change the firing of GnRH neurons

**Figure 7.**
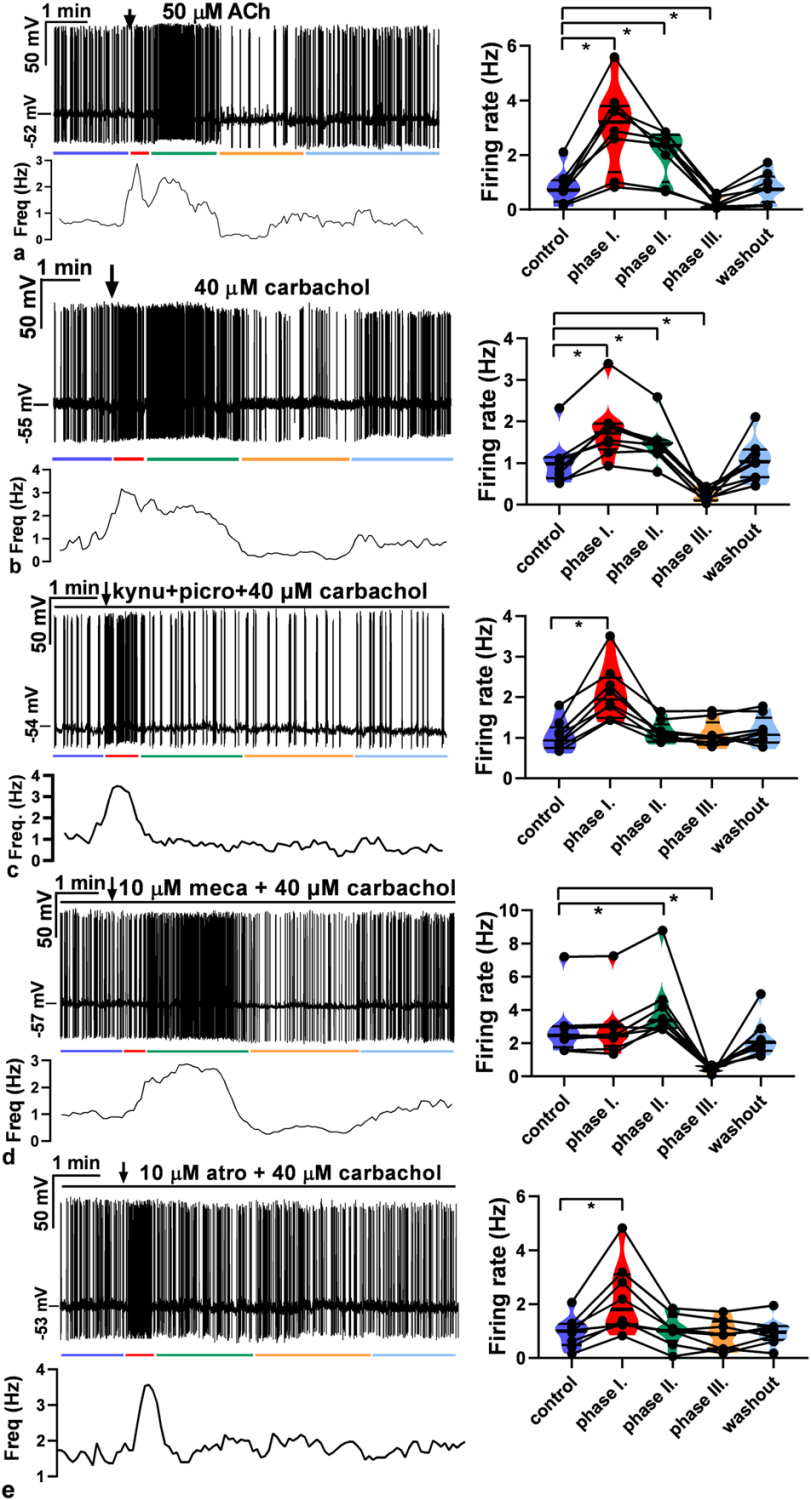
Effect of ACh and carbachol on the firing rate of GnRH neurons. ***a***, ACh (50  µM) evoked a biphasic action. First, it increased and then robustly inhibited the firing rate. ***b***, Carbachol (40  µM) also triggered a biphasic effect with a first elevation followed by a later decline in the firing rate. ***c***, In the presence of the GABA_A_-R inhibitor picrotoxin and the glutamate-R blocker kynurenic acid, carbachol induced a brief transient increase in the firing rate. ***d***, Mecamylamine (10  µM, nAChR antagonist) pretreatment eliminated the first, 0.5 min part of the elevation phase, suggesting that the facilitatory phase can be further divided into two subphases standing for nicotinic (phase I) and muscarinic (phase II) stimulatory actions, respectively. ***e***, Atropine (10 µM) pretreatment abolished both phase II of facilitation and the inhibitory phase (phase III), proving that these phases stand for muscarinic effects. The arrow shows the time of administration of ACh or carbachol. The horizontal black line above the recordings indicates the presence of inhibitors (picrotoxin + kynurenic acid, mecamylamine or atropine). Changes in the firing rate of individual GnRH neurons are shown beside each recording. Frequency distribution of firing rate is graphed under each recording. Phase I, 0.5 min; phase II, 3 min; phase III, 3 min. The colored lines under the recordings match the colors of the violin graphs and represent the various phases. *p < 0.05, picro, picrotoxin; kynu, kynurenic acid; meca, mecamylamine; atro, atropine. Details of the statistical analysis are provided in Extended Data Figure 7-1.

PSCs and firing activity of GnRH neurons positively correlate with each other (Chu and Moenter, 2005; Christian and Moenter, 2007). To evaluate the effect of ACh on the electric activity of GnRH neurons, firing was recorded in whole-cell current-clamp mode. A single bolus of ACh (50 µM), the natural ligand of the cholinergic system, evoked a biphasic change in the firing rate (Fig. 7*a*). An initial stimulatory phase was followed by a robust inhibitory phase. A thorough examination of the recording, however, revealed that the stimulatory phase can be further divided into two subphases (phase I, 0.5 min; phase II, 3 min), while no subphases could be defined in the inhibitory phase (phase III, 3 min). The length of these phases was defined by an approximate estimation and did not take into consideration accidental overlaps. The firing rate changed significantly in all three phases (Fig. 7*a*; in Hz: control, 0.86 ± 0.22; phase I, 3.0 ± 0.55; phase II, 2.1 ± 0.31; phase III, 0.28 ± 0.080; washout, 0.84 ± 0.19).

10.1523/JNEUROSCI.1780-23.2024.f7-1 Figure 7-1 Two-way ANOVA and Tukey’s post-hoc tests of firing rate data in Fig. 7. Download Figure 7-1, DOCX file.

We also studied the effects of carbachol (a widely used AChR agonist not metabolized by ACh-esterase) administration (40 µM) on the firing rate of GnRH neurons (Fig. 7*b*). The phases of the evoked effects mimicked those of ACh and showed significant changes in the firing rate (Fig. 7*b*; in Hz: control, 1.0 ± 0.20; phase I, 1.8 ± 0.26; phase II, 1.5 ± 0.18; phase III, 0.23 ± 0.053; washout, 1.1 ± 0.19).

To provide evidence for the direct effect of carbachol on GnRH neurons, we measured firing rate in the presence of the GABA_A_-R inhibitor picrotoxin and the glutamate-R blocker kynurenic acid. A significant brief transient increase in the firing rate was observed (Fig. 7*c*; in Hz: control, 1.1 ± 0.13; phase I, 2.1 ± 0.24; phase II, 1.2 ± 0.09; phase III, 1.1 ± 0.11; washout, 1.2 ± 0.12).

To elucidate the nature of AChRs involved in the observed effect, we pretreated the brain slices with mecamylamine (10 µM) and then applied carbachol. Carbachol application first accelerated and then inhibited the firing activity of GnRH neurons (Fig. 7*d*). The examination of the recordings, however, clearly showed that phase I was absent, whereas phase II was present in the stimulatory phase, showing that functional nAChRs play a role in phase I. Statistical analysis revealed that phase II and phase III represented significant changes in the firing rate (Fig. 7*d*; in Hz: control, 2.9 ± 0.64; phase I, 2.9 ± 0.65; phase II, 4.1 ± 0.71; phase III, 0.44 ± 0.069; washout, 2.3 ± 0.42), suggesting that mAChRs also take part in the cholinergic control of GnRH neuron firing.

To confirm the contribution of mAChRs to the control of GnRH neuron firing, we pretreated the brain slices with atropine (10 µM) and then added carbachol in a single bolus to the aCSF in the measuring chamber. The recordings showed that carbachol induced a short stimulatory phase, but no subsequent inhibition occurred in the presence of atropine (Fig. 7*e*). The short stimulation resembled phase I of Figure 7*b* and *c*. In contrast, phases II and III were absent (Fig. 7*e*; in Hz: control, 0.96 ± 0.21; phase I, 2.2 ± 0.47; phase II, 1.0 ± 0.20; phase III, 0.86 ± 0.20; washout, 0.97 ± 0.18).

Altogether, these results show that nAChRs control phase I of the evoked firing, while phases II and III are regulated by stimulatory and inhibitory subtypes of mAChRs, respectively.

### Electrophysiological characterization of functional nicotinic receptor subtypes involved in the control of GnRH neurons

**Figure 8.**
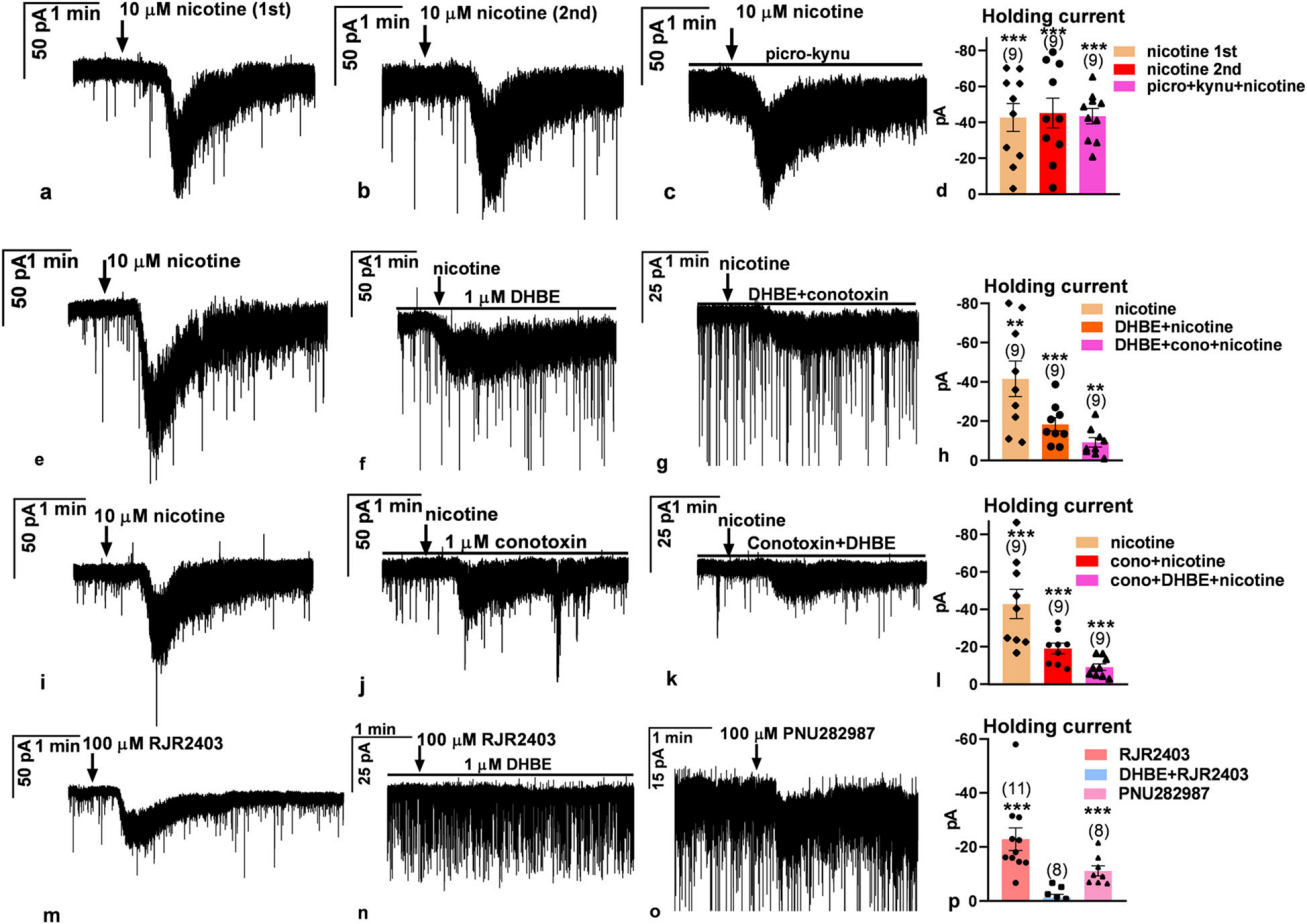
Characterization of functional nAChRs in GnRH neurons. Representative whole-cell patch voltage-clamp recordings demonstrate that nicotine evokes an inward current in GnRH neurons, depending on the specific type of the ionotropic AChR. ***a***, Nicotine (10  µM) triggered a robust inward current. ***b***, A second application of nicotine to the same neuron as in ***a*** evoked a similar response. ***c***, Nicotine application in the presence of picrotoxin and kynurenic acid (blocking the major synaptic inputs) triggered an inward current. ***d***, The bar graph summarizes these results. ***e***, In another neuron, nicotine administration induced an inward current. ***f***, The α4β2 nAChR antagonist, DHBE (1 µM), significantly reduced the nicotine-evoked inward current measured in the same neuron as in ***e***. ***g***, The coapplication of the α3β4 antagonist conotoxin (1 µM) with DHBE dampened the nicotine-evoked inward current further but did not eliminate it. The recording was carried out in the same neuron as in ***e*** and ***f***, showing that neurons expressing the α4β2 receptor also present the α3β4 receptor. ***h***, The bar graph summarizes the significant inward currents measured. ***i***, In another neuron, nicotine also induced a strong inward current. ***j***, The α3β4 receptor antagonist, conotoxin (1 µM), significantly reduced the evoked inward current measured in the same neuron as in ***i***. ***k***, The coadministration of DHBE attenuated the inward current further but did not abolish it. The recording was measured in the same neuron as in ***i*** and ***j***, showing that neurons expressing the α3β4 receptors also have functional α4β2 receptors. ***l***, The bar graph summarizes these results. ***m***, The α4β2 receptor agonist, RJR2403 (100 µM), also triggered an inward current which was abolished by DHBE pretreatment (***n***). ***o***, The α7 subtype nAChR agonist, PNU282987 (100 µM), also evoked a low-amplitude inward current. ***p***, The bar graph summarizes these results. DHBE, dihydro-β-erythroidine hydrobromide (α4β2 antagonist); conotoxin, α-conotoxin AuIB (α3β4 antagonist); RJR2403 (α4β2 agonist); PNU282987 (α7 agonist); picro, picrotoxin (GABA_A_-R antagonist); kynu, kynurenic acid (glutamate-R antagonist). The arrow shows the time of administration of various drugs. The horizontal line above the recordings shows continuous presence of the various inhibitors. *p < 0.05, **p < 0.01, ***p < 0.005. The recordings in ***e–g*** were carried out in a slice, and the recordings in ***i–k*** were carried out in another slice, respectively. Details of the statistical analysis are provided in Extended Data Figure 8-1 and 8-2.

The qRT-PCR study revealed the expression of genes encoding various subunits of nAChR in GnRH neurons. Whole-cell voltage-clamp measurements were carried out to identify the functional subtypes of nAChR involved in the control of GnRH neurons. Application of nicotine (10 µM, broadband agonist of nAChRs) triggered a robust inward current (amplitude, −45.2 ± 8.26 pA; duration, 51.3 ± 16.9 s; Fig. 8*a*). One minute after the inward current had decayed, nicotine was applied again (Fig. 8*b*). The amplitude and duration of the second response to nicotine were similar as in its first application (amplitude, −46.8 ± 9.12 pA; duration, 54.7 ± 19.1 s), indicating that desensitization does not occur at this time scale. Blockade of the major synaptic inputs by picrotoxin and kynurenic acid showed no effect on the evoked inward current, indicating a direct action of nicotine (amplitude, −42.6 ± 4.69 pA; duration, 48.2 ± 17.4 s; Fig. 8*c*). In another experiment after observing the inward current (amplitude, −41.5 ± 9.05 pA; duration, 58.9 ± 18.3 s; Fig. 8*e*,*h*), the slice was pretreated with dihydro-β-erythroidine hydrobromide (DHBE; 1 µM, antagonist of the α4β2 nAChR), and nicotine was applied again to the same neuron. An inward current was evoked again; however, its amplitude was significantly lower compared with the one observed when nicotine was added alone (amplitude, −18.4 ± 3.41 pA; duration, 84.4 ± 33.41 s; Fig. 8*f*,*h*), indicating that the α4β2 subtype of nAChR plays a role in the observed effect. Next, α-conotoxin AuIB (1 µM, antagonist of α3β4 nAChR) was added to the aCSF containing DHBE, and nicotine was administered again to the same neuron. This inhibitor cocktail significantly decreased the amplitude further (amplitude, −9.2 ± 2.36 pA; duration, 78.2 ± 38.6 s; Fig. 8*g*,*h*), although it did not abolish the response completely. This suggests that GnRH neurons that express the α4β2 subtype also present the α3β4 subtype of nAChR.

10.1523/JNEUROSCI.1780-23.2024.f8-1 Figure 8-1 One-way ANOVA of amplitudes of the inward currents in Fig. 8. Download Figure 8-1, DOCX file.

10.1523/JNEUROSCI.1780-23.2024.f8-2 Figure 8-2 Two-tailed Student’s t-test of amplitudes of inward currents in Fig. 8. Download Figure 8-2, DOCX file.

To confirm this coexpression further, we next examined whether GnRH neurons expressing the α3β4 nAChR subtype also express the α4β2 subtype. Therefore, after measuring the nicotine-evoked inward current (amplitude, −42.9 ± 7.89 pA; duration, 68.7 ± 21.3 s; Fig. 8*i*,*l*), α-conotoxin AuIB was first added to the aCSF, and the nicotinic response was recorded again in the same neuron. Conotoxin pretreatment significantly decreased but did not eliminate the amplitude of the inward current (amplitude, −19.2 ± 2.83 pA; duration, 74.1 ± 28.39 s; Fig. 8*j*,*l*), showing expression of the functional α3β4 type receptor. Then DHBE was also added to the aCSF. This cocktail significantly lowered the amplitude further but did not abolish the evoked response (amplitude, −9.2 ± 1.74 pA; duration, 71.1 ± 26.98 s; Fig. 8*k*,*l*).

To present further evidence that the α4β2 subtype of nAChR is expressed in GnRH neurons, we applied RJR2403 (100 µM, selective agonist of the α4β2 receptor) in a single bolus. Application of RJR2403 evoked an inward current (amplitude, −22.9 ± 4.15 pA; duration, 98.9 ± 38.15 s; Fig. 8*m*,*p*), which was abolished completely by DHBE pretreatment (amplitude, −1.2 ± 1.23 pA; Fig. 8*n*,*p*). Because simultaneous blockade of α4β2 and α3β4 subtypes of nAChR did not completely eliminate the nicotine-evoked response in GnRH neurons, the participation of further nicotinic receptor(s) seemed reasonable in the regulatory process. We challenged the putative expression of the α7 subtype of the receptor. Therefore, PNU282987 (100 µM, selective agonist of the α7 subunits) was applied into the bath in a single bolus. It evoked a low amplitude but still significant inward current (amplitude, −11.2 ± 1.88 pA; duration, 88.6 ± 25.15 s; Fig. 8*o*,*p*). This finding indicates that the α7 subtype of nAChR also contributes to the regulation of GnRH neurons, in addition to the α4β2 and the α3β4 receptor subtypes.

### Characterization of muscarinic receptor subtypes controlling GnRH neuron activity

#### Regulation of mPSC frequencies

**Figure 9.**
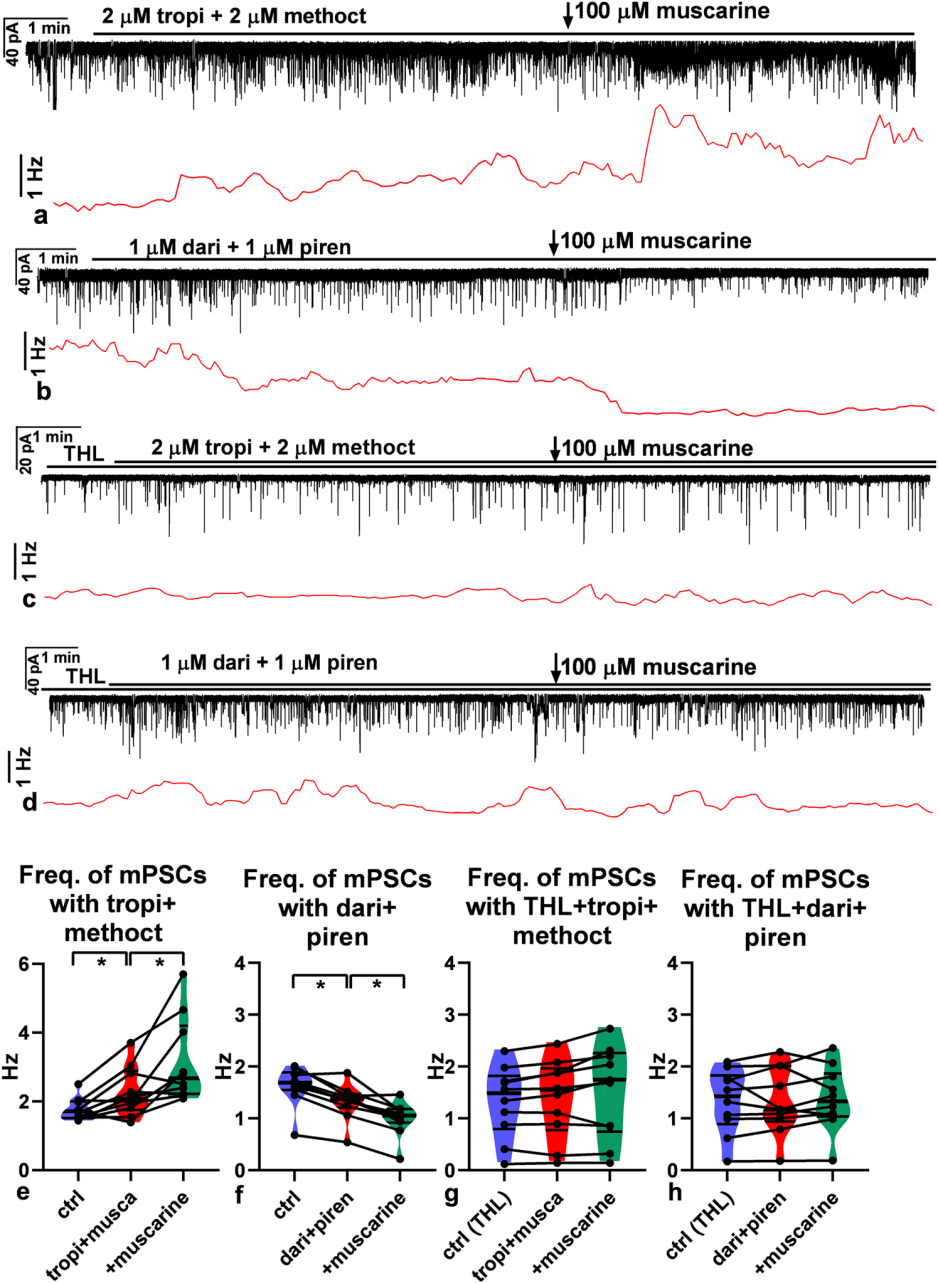
Functional characterization of mAChRs in GnRH neurons. Representative recordings prove the effect of blockade of inhibitory (tropicamide + methoctramine) or stimulatory (darifenacin + pirenzepine) muscarinic receptors and muscarine on mPSCs in GnRH neurons in the intracellular absence or presence of THL, an inhibitor of retrograde 2-AG endocannabinoid signaling pathway. ***a***, Blockade of inhibitory receptors increased frequency of mPSCs. Application of muscarine elevated the frequency further. ***b***, Conversely, blockade of stimulatory receptors diminished the frequency of mPSCs, and muscarine reduced it further. ***c***, ***d***, Intracellular presence of THL, however, abolished the effects; neither blockade of inhibitory and stimulatory receptors nor application of muscarine evoked any significant action on the frequency. ***e–h***, The graphs summarize these significant actions and their elimination in the presence of THL. Horizontal black lines above the recordings show the presence of various inhibitors [THL; dari, darifenacin (M3 inhibitor); methoct, methoctramine (M2 inhibitor); piren, pirenzepine (M1 inhibitor); tropi, tropicamide (M4 inhibitor)] and arrow points the time of muscarine administration). ctrl, 1 min long period before any inhibitor is applied in ***a***, ***b***, ***e***, and ***f***; ctrl(THL), 1 min long period when THL is present in the aCSF but no other inhibitors in ***c***, ***d***, ***g***, and **h**. *p < 0.05. Frequency distribution graphs are under each recording. Details of the statistical analysis are provided in Extended Data Figure 9-1.

Using whole-cell voltage-clamp recordings, we examined the contribution of stimulatory (M1, M3) and inhibitory (M2, M4) muscarinic receptors to the regulation of mPSC frequencies in GnRH neurons. We have to note that mPSCs are exclusively GABAergic (Bhattarai et al., 2011) and GABA is excitatory via GABA_A_-R due to the high intracellular chloride concentration in GnRH neurons of male mice (Herbison and Moenter, 2011). Following a 1-min-long control registration period, blockade of the inhibitory receptors was carried out with a cocktail consisting of the M2 and M4 muscarinic receptor inhibitors, tropicamide and methoctramine, respectively. The treatment significantly increased the frequency of the mPSCs, suggesting a tonic release of ACh (Fig. 9*a*,*e*). Then, muscarine (100 µM) was added in a single bolus into the measuring chamber in the continuous presence of the blocker cocktail. Muscarine significantly increased the frequency further (Fig. 9*a*,*e*; in Hz: control, 1.8 ± 0.10; tropi + methoct cocktail, 2.3 ± 0.23; cocktail + musca, 3.1 ± 0.39). In the second step, the M1 and M3 stimulatory receptors were antagonized with a cocktail of darifenacin and pirenzepine, inhibitors of the M1 and M3 muscarinic receptors, respectively, and the measurements were carried out again. Blockade of the stimulatory muscarinic receptors decreased the frequency of the mPSCs significantly, confirming the tonic release of ACh (Fig. 9*b*,*f*). Muscarine administration significantly diminished the frequency further (Fig. 9*b*,*f*; in Hz: control, 1.6 ± 0.12; dari + piren cocktail, 1.3 ± 0.11; cocktail + musca, 0.99 ± 0.10).

10.1523/JNEUROSCI.1780-23.2024.f9-1 Figure 9-1 Two-way ANOVA and Tukey’s post-hoc tests of mPSC frequency data in Fig. 9. Download Figure 9-1, DOCX file.

Our earlier studies revealed that activation of retrograde 2-AG endocannabinoid signaling pathways can change the frequency of mPSCs in GnRH neurons. Therefore, THL, an inhibitor of 2-AG endocannabinoid production, was applied intracellularly in the patch pipette solution. Under this condition, a cocktail of tropicamide and methoctramine evoked no notable change in the frequency of the mPSCs (Fig. 9*c*,*g*), and muscarine application triggered no observable change either (Fig. 9*c*,*g*; in Hz: THL control, 1.3 ± 0.22; THL + tropi + methoct cocktail, 1.4 ± 0.24; cocktail + musca, 1.5 ± 0.28). Similarly, a cocktail of darifenacin and pirenzepine resulted in no notable change in the frequency in the intracellular presence of THL (Fig. 9*d*,*h*) and muscarine induced no significant alteration of the frequency either (106.4 ± 4.97% of the control value; Fig. 9*d*,*h*; in Hz: THL control, 1.3 ± 0.20; THL + dari + piren cocktail, 1.3 ± 0.20; cocktail + musca, 1.4 ± 0.20). These data unveil the regulatory role of retrograde 2-AG endocannabinoid signaling in the modulation of GnRH neuron physiology via both inhibitory and stimulatory types of muscarinic receptors. In addition, the presence of this retrograde machinery explains why firing rate presented no change in phases II and III when the major synaptic inputs to GnRH neurons were blocked (Fig. 7*c*).

#### Muscarine exerts a biphasic effect on firing of GnRH neurons

**Figure 10.**
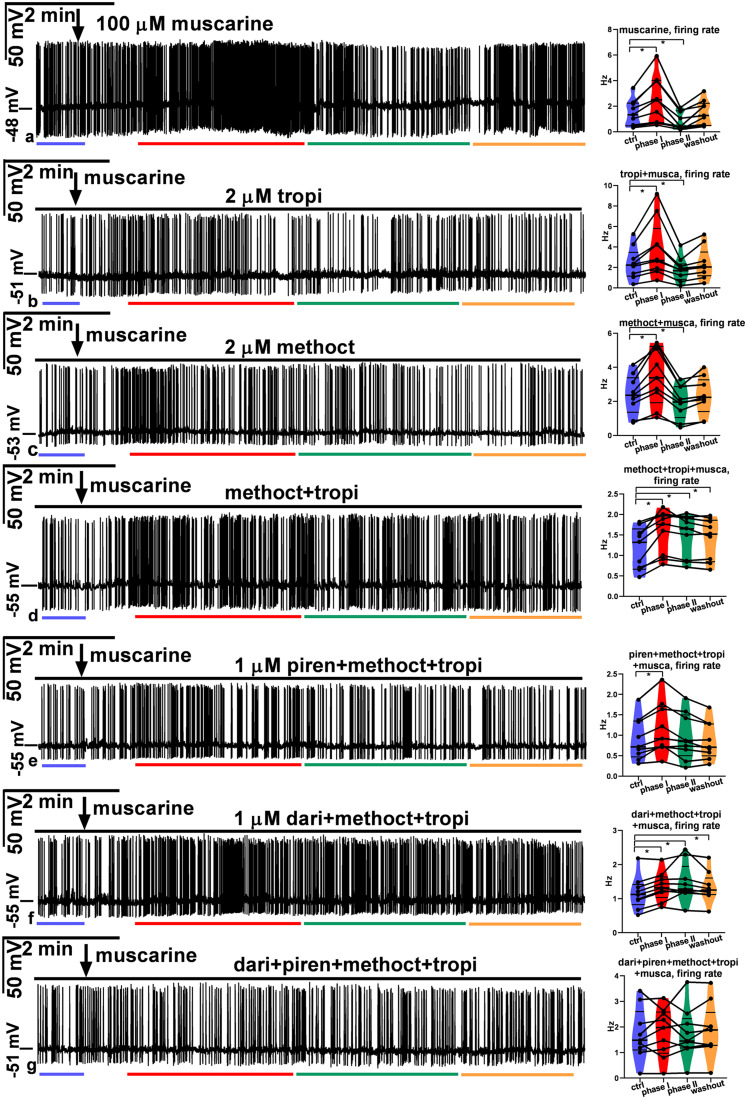
Impact of muscarine receptor subtypes on firing of GnRH neurons. Representative whole-cell patch current-clamp recordings show that muscarine evoked a biphasic effect on the firing of the GnRH neurons expressing various muscarine receptor subtypes. ***a***, In the first, ∼2.5 min period, muscarine (100 µM) enhanced (phase I) the firing rate of the GnRH neuron followed by a decreased activity period (phase II). ***b***, The inhibitory M4-muscarine receptor antagonist tropicamide (2 µM) dampened the inhibitory effect of muscarine (as seen in phase II). ***c***, The inhibitory M2-muscarine receptor antagonist methoctramine (2 µM) also decreased the inhibitory effect of muscarine (phase II). ***d***, Coadministration of tropicamide and methoctramine eliminated the inhibitory effect of muscarine (phase II). ***e***, The stimulatory M1-muscarine receptor antagonist, pirenzepine (1 µM), dampened the stimulatory effect of muscarine (as seen in phase I) in the presence of the M2 and M4 antagonists. ***f***, The stimulatory M3-muscarine receptor antagonist, darifenacin (1 µM), also attenuated the stimulatory effect of muscarine (phase I). ***g***, Neither facilitation nor inhibition was seen when a cocktail of all four antagonists was applied. The black line above each recording shows the presence of cocktails of various muscarine receptor subtype inhibitors in the aCSF (dari, darifenacin; methoct, methoctramine; piren, pirenzepine; tropi, tropicamide). The colored lines under each recording present periods of the control (ctrl, blue), the stimulatory phase (phase I, red), the inhibitory phase (green, phase II), and the washout (orange). The graphs beside each recording summarize the effect of muscarine in the presence of the various cocktails of M1–M4 inhibitors (the colors match the colors of the horizontal lines representing the phases). Arrows show the administration of muscarine. *p < 0.05. Details of the statistical analysis are provided in Extended Data Figure 10-1.

Our results suggested that besides the nAChRs, functional mAChR subtypes also exist in GnRH neurons. Therefore, the putative effects of the M1–M4 receptor forms on the firing activity of GnRH neurons were studied using the whole-cell current-clamp method. When muscarine (100 µM, broadband agonist of mAChRs) was administered in a single bolus, it evoked a biphasic effect. In the first 3 min period (phase I), muscarine significantly elevated the firing rate (Fig. 10*a*). In the second 3 min period (phase II), a significant decrease was seen in the firing rate which was then washed out (Fig. 10*a*; in Hz: control, 1.5 ± 0.35; phase I, 2.5 ± 0.62; phase II, 0.85 ± 0.24; washout, 0.85 ± 0.24). To further dissect the phenomenon, first, we examined the function of M4 by pretreating the brain slices with 2 µM tropicamide, a selective inhibitor of the M4 subtype of the mAChR, and then administered muscarine. The recording clearly showed that both the stimulatory and inhibitory phases developed (Fig. 10*b*). The firing rate significantly increased and then significantly decreased (in Hz: control, 2.4 ± 0.51; phase I, 3.9 ± 0.94; phase II, 1.8 ± 0.39; washout, 2.4 ± 0.52). However, phase II represented a decrease that was milder than without tropicamide, suggesting that, beside M4, another inhibitory subtype is involved. Next, M2 was blocked by adding 2 µM methoctramine, a selective inhibitor of the M2 subtype of mAChR, and then muscarine was applied. The stimulatory phase I persisted in the presence of methoctramine, revealing a significant increase in the firing rate (Fig. 10*c*). The inhibitory phase II was also present because the firing rate significantly diminished (Fig. 10*c*; in Hz: control, 2.4 ± 0.38; phase I, 3.5 ± 0.56; phase II, 2.0 ± 0.33; washout, 2.3 ± 0.36). Phase II, however, displayed a smaller decrease than in the absence of the M2 or M4 antagonists, revealing the role of the M2 receptor subtype.

10.1523/JNEUROSCI.1780-23.2024.f10-1 Figure 10-1 Two-way ANOVA and Tukey’s post-hoc tests of firing rate data in Fig. 10. Download Figure 10-1, DOCX file.

Next, we antagonized both inhibitory subtypes. In the presence of a cocktail of tropicamide and methoctramine, the stimulatory phase I still existed, but no inhibitory phase II developed; rather, stimulation was seen in phase II period (Fig. 10*d*; in Hz: control, 1.2 ± 0.17; phase I, 1.6 ± 0.18; phase II, 1.5 ± 0.18; washout, 1.4 ± 0.17). The changes in both phases were significant. The stimulatory period, therefore, lasted much longer in the presence of the M2 and M4 antagonists.

We also examined the function of the stimulatory mAChR subtypes, M1 and M3. First, pirenzepine (1 µM, selective antagonist of the M1 subtype of mAChR) was added to the cocktail of tropicamide and methoctramine, and then muscarine was applied. In the presence of these three antagonists, muscarine significantly increased the firing rate in phase I (Fig. 10*e*). Nevertheless, the value of the increase was lower than that without pirenzepine, showing the role of M1 in the stimulation. Phase II, however, presented no significant change at all (in Hz: control, 0.91 ± 0.17; phase I, 1.2 ± 0.21; phase II, 0.95 ± 0.19; washout, 0.87 ± 0.15). Thus, the duration of the stimulation was not longer than 3 min.

The role of M3, the other stimulatory mAChR subtype, was also investigated. Darifenacin (1 µM, selective antagonist of the M3 subtype of mAChRs) was added to the cocktail of tropicamide and methoctramine, and then muscarine was applied. Phase I presented a significant elevation in the firing rate (Fig. 10*f*). Phase II also displayed a significant increase in the firing rate (Fig. 10*f*; in Hz: control, 1.2 ± 0.16; phase I, 1.4 ± 0.14; phase II, 1.5 ± 0.19; washout, 1.3 ± 0.15), thus the stimulation lasted much longer than 3 min.

Finally, the effect of muscarine was examined in the presence of M1–M2–M3–M4 antagonists. This cocktail abolished both phases I and II completely (Fig. 10*g*; in Hz: control, 1.7 ± 0.34; phase I, 1.8 ± 0.32; phase II, 1.7 ± 0.33; washout, 1.9 ± 0.35).

### Optogenetic activation of cholinergic afferents changes the electrophysiological activity of GnRH neurons and reveals cotransmission of ACh and GABA

**Figure 11.**
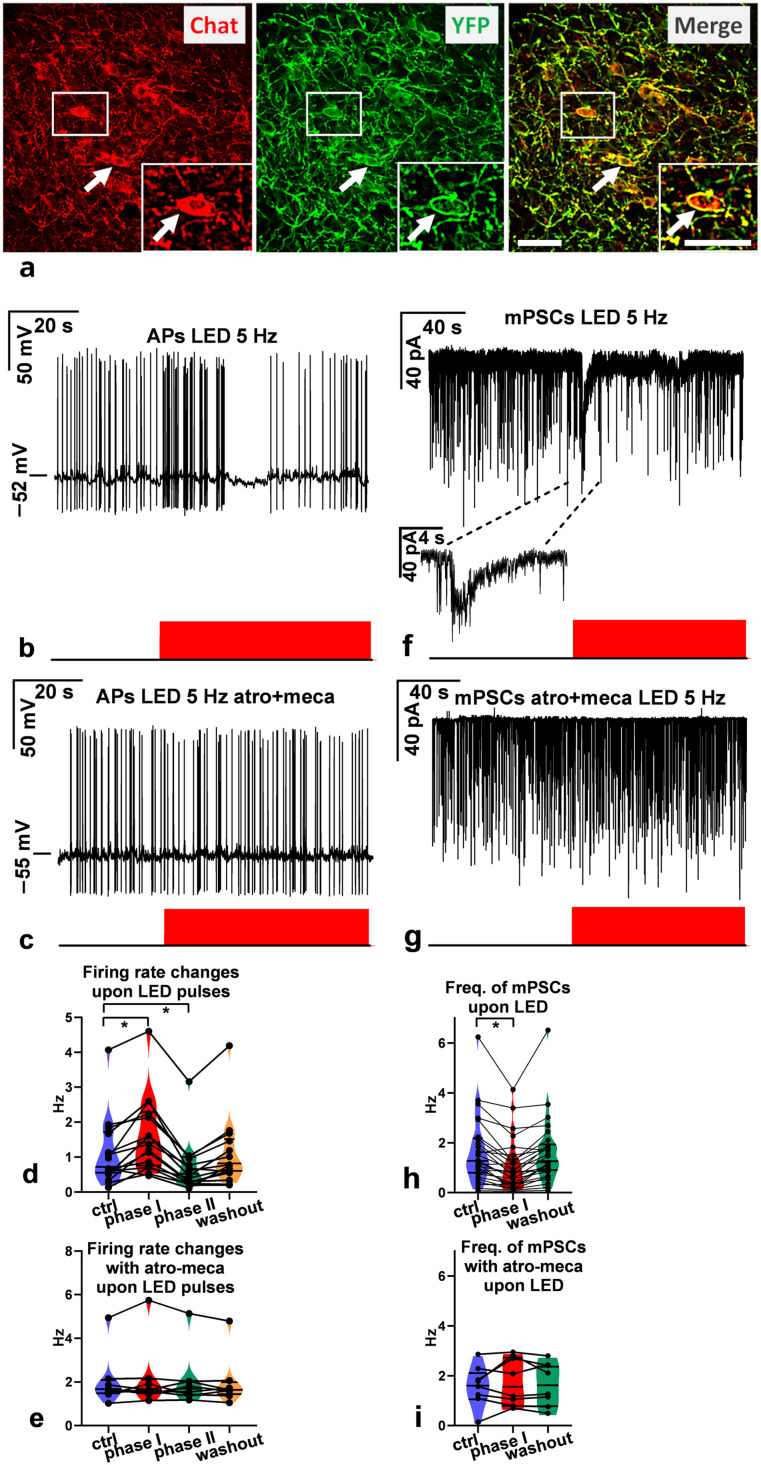
Effects of optogenetic stimulation of cholinergic axons upon firing activity and frequency of spontaneous mPSCs of GnRH neurons. ***a***, Expression of ChR2-eYFP in cholinergic neurons of the MS in the Chat-ChR2-eYFP transgenic mouse. Cholinergic neurons immunolabeled for choline acetyltransferase (Chat, red) are also immunopositive for eYFP (green) demonstrating the coexpression of Chat and eYFP IRs (Merge). Arrows point to identical neurons. Scale bar, 25 µm. ***b***, LED illumination at 5 Hz evoked an initial facilitation followed by transient inhibition in firing. ***c***, Both the stimulatory and inhibitory effects were eliminated when the slices were pretreated with the cocktail of atropine and mecamylamine. ***d***, ***e***, The graphs summarize the effect of LED illumination at 5 Hz, revealing that ACh was released from channelrhodopsin-enriched cholinergic terminals. ***f***, LED illumination at 5 Hz triggered an inward current, followed by a transient decrease in the mPSC frequency. The inset is a zoomed-in region of the graph containing the inward current. ***g***, Both the inward current and the diminished mPSC frequency disappeared in the presence of a cocktail of atropine and mecamylamine. ***h***, ***i***, The graphs show a significant difference in the mPSC frequencies measured in the absence or presence of the inhibitor cocktail. Atro, atropine; meca, mecamylamine. Recordings are in black; red bars underneath the recordings indicate the period of LED illumination. *p < 0.05. Details of the statistical analysis are provided in Extended Data Figure 11-1 and 11-2.

To study the effect of endogenous ACh, we examined GnRH neurons of the triple-transgenic Chat-Cre-ChR2-GnRH-GFP mice in acute brain slices using LED square pulses (470 nm, 5 ms) to activate channelrhodopsin-expressing cholinergic axons. In this transgenic mouse line, ChR2 was expressed exclusively in cholinergic neurons (Fig. 11*a*), including their axons. GnRH neurons were whole-cell clamped in current-clamp mode at 0 pA to detect firing. After a 30 s control period, an LED train of 5 Hz was applied for a subsequent 60 s while firing was continuously recorded. A biphasic pattern was detected when a 5 Hz train was applied: the initial stimulation was followed by a transient inhibition (in Hz: control, 1.5 ± 0.35; phase I, 2.5 ± 0.62; phase II, 0.85 ± 0.24; washout, 1.5 ± 0.32). Stimulation lasted for 15 ± 2.7 s; inhibition lasted for 38 ± 3.4 s (Fig. 11*b*,*d*). Both stimulation and inhibition disappeared in the presence of a cocktail of atropine and mecamylamine (Fig. 11*c*,*e*; in Hz: control, 2.4 ± 0.51; phase I, 3.9 ± 0.94; phase II, 1.8 ± 0.39; washout, 2.4 ± 0.52), suggesting the involvement of ACh in the process.

10.1523/JNEUROSCI.1780-23.2024.f11-1 Figure 11-1 Two-way ANOVA and Tukey’s post-hoc tests of firing rate data in Fig. 11. Download Figure 11-1, DOCX file.

10.1523/JNEUROSCI.1780-23.2024.f11-2 Figure 11-2 Two-way ANOVA and Tukey’s post-hoc tests of mPSC frequency data in Fig. 11. Download Figure 11-2, DOCX file.

The effects of optogenetic activation of cholinergic afferents were seen in the membrane currents, too. After a 2 min control period, LED illumination at 5 Hz for 2 min induced an initial inward current (amplitude, 51 ± 12.7 pA; Fig. 11*f*) resembling those triggered by carbachol. A transient decrease in the frequency was then observed (Fig. 11*f*,*h*; in Hz: control, 1.9 ± 0.48; phase I, 1.0 ± 0.20; washout, 1.8 ± 0.44). Both the inward current and the decreased frequency were eliminated in the presence of atropine + mecamylamine (Fig. 11*g*,*i*; in Hz: control, 1.6 ± 0.29; phase I, 1.9 ± 0.40; washout, 1.7 ± 0.31).

**Figure 12.**
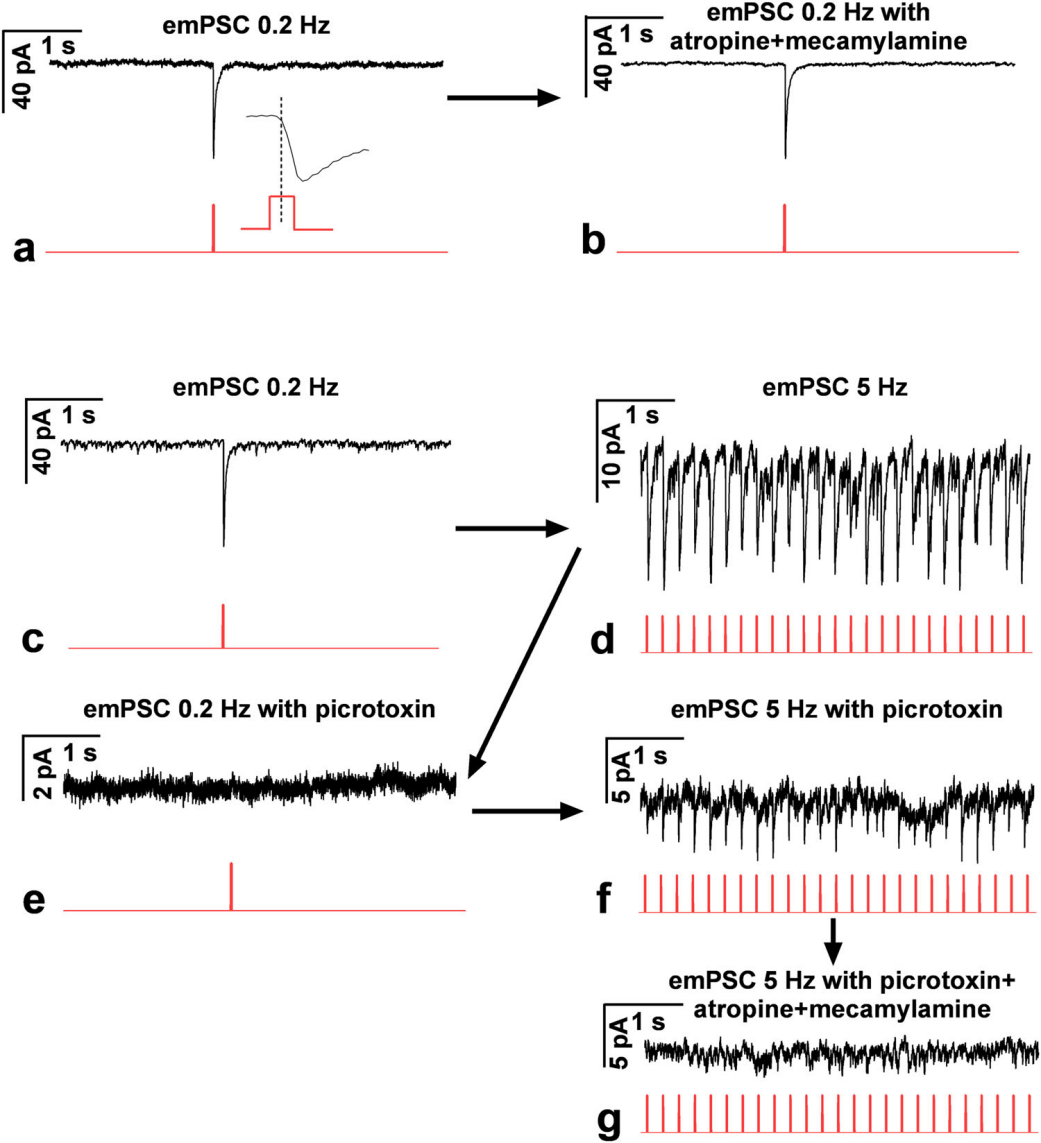
emPSCs observed in a subpopulation of GnRH neurons of Chat-Cre-ChR2-GnRH-GFP transgenic mice upon LED illumination at 0.2 or 5 Hz. ***a***, A robust inward emPSC was triggered by LED illumination at 0.2 Hz. ***b***, The emPSC was still seen in the presence of atropine and mecamylamine in the same neuron as in ***a***. ***c***, A similar emPSC was revealed in another GnRH neuron at 0.2 Hz. ***d***, A train of emPSCs of lower amplitude was evoked by LED at 5 Hz in the same neuron as in ***c***. ***e***, The emPSC observed at 0.2 Hz was eliminated by picrotoxin in the same neuron as in ***d***. ***f***, A train of emPSCs was still detected in the presence of picrotoxin at 5 Hz in the same neuron as in ***e***. ***g***, This train was abolished by adding atropine and mecamylamine to the picrotoxin-containing aCSF in the same neuron as in ***f***. Arrows show the time order of measurements in the same neuron. Recordings are in black; red lines underneath the recordings indicate the period of LED illumination at 0.2 or 5 Hz, respectively.

To examine the extrasynaptic and/or synaptic character of cholinergic neurotransmission onto GnRH neurons, we investigated the existence or absence of evoked mPSC (emPSC) in the presence of TTX in GnRH neurons of the adult male Chat-Cre-ChR2-GnRH-GFP transgenic mice. LED illumination (0.2 Hz, 470 nm, 5 ms duration, then averaged of 60 runs of the 5-s-long recordings) triggered no visible emPSC response in 90% of GnRH neurons suggesting an extrasynaptic action of ACh. In 10% of the cells, however, robust emPSC was seen (Fig. 12*a*). The emPSCs occurred synchronously with the LED square pulse (Fig. 12, zoomed inset). The onset was 2–3 ms after the rise of the illumination. The emPSC was still observable in the same GnRH neuron in the presence of atropine and mecamylamine (10–10 µM; Fig. 12*b*) showing the release of a neurotransmitter or neuromodulator other than ACh.

In other GnRH neurons of the triple transgenic mouse in which emPSC was evoked by LED of 0.2 Hz (Fig. 12*c*), we examined the effect of a 5 Hz LED illumination (5 Hz train, then averaged of 60 runs of the 5-s-long recordings). In 50% of these neurons, a train of emPSCs was seen (Fig. 12*d*). These emPSCs were synchronous with the 5 Hz LED pulses, although the amplitude was lower than the one evoked by the 0.2 Hz (≍20%). To identify the transmitter(s) responsible for the emPSCs, first we added picrotoxin (100 µM) to the aCSF and examined the existence or absence of synaptic response at 0.2 or 5 Hz in the same GnRH neuron as in Fig. 12*c*,*d*. Picrotoxin abolished the emPSC at 0.2 Hz completely, providing strong evidence for the exclusive release of GABA from ChR2-tagged cholinergic axons (Fig. 12*e*) at this LED frequency. Surprisingly, a train of emPSCs was still detected at 5 Hz illumination (Fig. 12*f*) in the presence of picrotoxin in this same neuron. The amplitudes of these emPSCs were lower than those detected without the GABA_A_-R blocker. The emPSCs triggered by LED at 5 Hz were eliminated when atropine–mecamylamine was added to the aCSF beside picrotoxin (Fig. 12*g*). The last two observations suggest the release of both ACh and GABA at 5 Hz. These results show that cholinergic axons innervating GnRH neurons use ACh and GABA for neurotransmission in a frequency-dependent manner.

**Figure 13.**
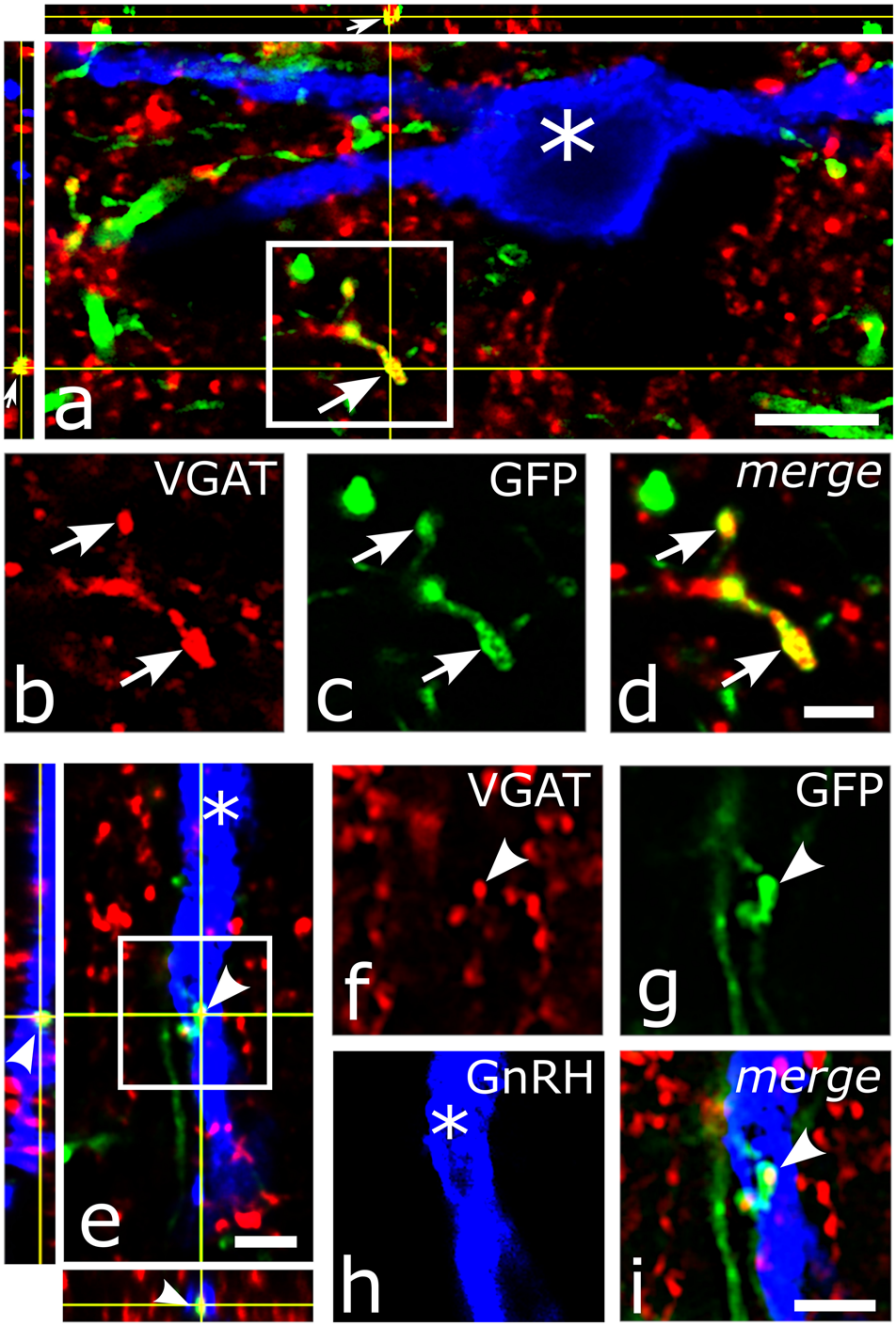
Expression of VGAT in cholinergic axons targeting GnRH neurons in the Chat-Cre-ChR2 mouse. ***a***, Triple immunofluorescent imaging revealed VGAT (red channel), ChR2-GFP (green), and GnRH (blue)-IR structures via confocal microscopy. VGAT (***b***) and ChR2-GFP (***c***) were colocalized (***d***) within the same axon varicosity (enframed in ***a***, enlarged in ***d***, arrows) near a GnRH neuron (blue, asterisk). The juxtaposition of a VGAT (***f***, arrowhead) and ChR2-GFP (***g***) IR double-labeled axon varicosity (***i***) with the dendrite of a GnRH neuron (***h***, asterisk) was confirmed in orthogonal views (***e***; enframed area enlargement in ***i***). Scale bars: ***a***, 5 µm; ***b–i***, 2 µm.

The above optogenetic studies showed that GABA and ACh are cotransmitted from a subpopulation of cholinergic axon terminals, depending on the frequency of LED stimulation. To provide the structural correlate of ACh/GABA cotransmission, we performed immunofluorescence triple labeling on preoptic sections of Chat-Cre-ChR2 transgenic mouse brains for VGAT, GFP-tagged channelrhodopsin-2 (ChR2-GFP), and GnRH. The confocal microscopic analysis revealed that VGAT-IR (Fig. 13*a*,*b*) and cholinergic ChR2-GFP-IR (Fig. 13*a*,*c*) axons exist in the proximity of GnRH neurons. At high power, the VGAT (Fig. 13*b*) and ChR2-GFP (Fig. 13*c*) were colocalized (Fig. 13*d*) in the same cholinergic axon varicosities. Furthermore, VGAT- (Fig. 13*f*) and ChR2-GFP–expressing (Fig. 13*g*) double-labeled axon boutons (Fig. 13*e*,*i*) were found in juxtaposition (Fig. 13*e*,*i*) with GnRH dendrite (Fig. 13*h*,*i*). These morphological results unveiled the dual ACh/GABA chemotype of at least a subset of cholinergic axons innervating GnRH neurons and support the optogenetic findings on ACh/GABA cotransmission in regulation of GnRH neurons.

## Discussion

Pharmacogenetic activation of the central cholinergic system rapidly increased both basal and mean LH levels with peaks at 30 min, followed by a prolonged return to the basal level. The amplitude and frequency of LH pulses did not change. CNO applied in a wide concentration range (0.3–5 mg/bw kg) had no effect on the mean LH levels, amplitude, and frequency of LH pulses (Coutinho et al., 2020; Dulka et al., 2020; Ivanova et al., 2021; Vanacker et al., 2021) or the LH surge (Wang et al., 2020) in wild-type or DREADD-negative transgenic mice under in vivo conditions supporting the view that CNO treatment has no off-target effect in wild-type or DREADD-negative mice. In our experiment, the entire cholinergic system was activated by CNO administration resulting in a robust surge release of LH. GnRH neurons were direct targets of cholinergic modulation, and it is feasible that some of their primary afferent systems that are regulated by cholinergic neurotransmission were also involved. In concert with our finding, cholinergic drugs targeting the hypothalamus have previously been shown to increase the secretion of LH (Fiorindo and Martini, 1975). Furthermore, atropine administration blocked the proestrus surge of LH (Libertun and McCann, 1973).

GnRH neurons (Campbell et al., 2022) are surrounded by clusters of ACh-synthesizing neurons in the MS and the DBB (Wu et al., 2014; Li et al., 2018). The MS-DBB-mPOA region is rich in VAChT-IR axons (Schäfer et al., 1994). Our 3DISCO-based quantitative analysis unveiled that GnRH neurons are innervated by cholinergic axons. VAChT-IR axons were juxtaposed to dendrites and perikarya of GnRH neurons with a ∼6.4:1 ratio, emphasizing further the exquisite role of GnRH dendrites in communication with neurotransmitter systems (Norberg et al., 2013). Nevertheless, 37.4 ± 5.1% of GnRH-IR perikarya received cholinergic inputs. The ultrastructural analysis confirmed that VAChT and GnRH-IR profiles formed direct contacts without interposing astrocytic processes. The cholinergic terminals occasionally formed synapses with GnRH neurons in the MS-DBB-mPOA region, indicating sites of synaptic- and/or volume transmission (Sarter et al., 2009). A similar pattern of interaction was reported in rats (Turi et al., 2008). Cholinergic neurons distributed in the mouse brain (Li et al., 2018) project to the MS-DBB-mPOA. The retrograde viral labeling of cholinergic neurons wired to GnRH neurons revealed that cholinergic afferents of the GnRH neurons arise exclusively from the MS and DBB. MS was recently shown to provide cholinergic innervation of GnRH neurons (Shostak et al., 2023).

Regarding the subunit composition of nAChRs, the occurrence of α3β4, α4β2, and α7 receptors was confirmed in GnRH neurons. Expression of functional α4β2 and α7 receptors has previously been demonstrated (Huang et al., 2011) in the hypothalamus, and α3 subunit was detected in embryonic GnRH cells (Shostak et al., 2023). Interestingly, the α4, α7, β2, and β4 subunits were not found in the embryonic neurons, indicating different nAChR-mediated signaling in embryonic versus adult GnRH neurons. Our inward ion current measurements suggest the abundance of α4β2 and α3β4 receptors with a relatively low contribution of α7.

In terms of function, bath application of ACh or carbachol evoked a biphasic effect on GnRH neurons, first elevating and then decreasing their firing rate. Since electric activity and hormone release of GnRH neurons correlate (Ordög et al., 1997; Zhang et al., 2007; Pielecka-Fortuna et al., 2008), the result suggests that cholinergic drugs likely induce GnRH release. Similarly, ACh first activated embryonic GnRH neurons and increased their intracellular Ca^2+^ concentration, followed by a subsequent 5-min-long decline (Shostak et al., 2023). In our study, however, the first phase of the recordings was characterized by a fast transient increase followed by a slow, prolonged rise in the firing rate. Pretreatment of slices with nAChR antagonist mecamylamine eliminated the fast phase, leaving the slow-type facilitation and the inhibitory phase untouched, suggesting the role of nAChRs in the fast action. Indeed, nicotine can induce fast depolarization and enhance firing in orexin cells (Huang et al., 2011) and evoke GnRH release from hypothalamic fragments (Richardson et al., 1982). Mecamylamine did not affect slow facilitation and inhibition of firing, suggesting that they were due to the activation of mAChRs. Indeed, application of atropine abolished both slow components while the faster, transient facilitation remained intact. Goldfish (Kawai et al., 2013) and embryonic mouse GnRH neurons (Shostak et al., 2023) also respond to muscarine, supporting the current finding.

In our study, carbachol triggered an inward current, followed by a decrease in the frequency of mPSCs. Inward current indicates membrane depolarization, resulting in fast facilitation of the firing rate. The inward current was abolished by mecamylamine, suggesting the inevitable role of nAChRs in the process. In the hypothalamus, similar nAChR-dependent activation was reported in POMC neurons (Huang et al., 2011). Embryonic mouse GnRH neurons also responded to ACh administration with a nAChR-dependent fast and transient stimulatory change in membrane potential (Shostak et al., 2023) resembling the inward current we observed. The carbachol-triggered decrease in the frequency of mPSCs after the inward current was blocked by atropine indicates the involvement of mAChRs. mAChR-dependent ion currents were also observed in goldfish (Kawai et al., 2013) and embryonic mouse GnRH neurons (Shostak et al., 2023). Nicotine also triggered a robust inward ion current and excitation of GnRH neurons. The responses evoked by repeated application (90–120 s) of nicotine presented no desensitization at this period. Embryonic GnRH neurons process the ACh signal via α3, M2, and M4 receptors (Shostak et al., 2023) contrasting the wider set of AChRs we found in adults suggesting a major reshape of the AChR profile in GnRH neurons during differentiation.

We also demonstrated an inverse effect of muscarine on the mPSC frequency in the presence of either M2/M4 or M1/M3 receptor antagonists, which can be explained by the involvement of different intracellular signaling pathways. M1/M3 are G_q_-coupled receptors mediating neuronal excitation, whereas the G_i/o_-coupled M2/M4 activity leads to inhibition (Brown, 2018). The M2 or M4 antagonists prolonged the stimulatory phase, indicating that M2/M4 receptors can attenuate the cholinergic excitation. Both facilitatory and inhibitory actions were abolished by the intracellular application of THL which blocked synthesis and subsequent release of 2-AG from GnRH neurons. Involvement of retrograde 2-AG signaling in regulation of GnRH neurons was reported earlier (Farkas et al., 2010; Bálint et al., 2016, 2020; Farkas et al., 2016). Furthermore, the role of endocannabinoids in mAChR-mediated signal transduction was shown in other brain networks (Fukudome et al., 2004; Uchigashima et al., 2007; Martin et al., 2015; Joo et al., 2019; Tryon et al., 2023).

LED illumination of GnRH neurons at 5 Hz in Chat-Cre-ChR2-GnRH-GFP transgenic mice evoked a biphasic change in the firing rate which was abolished by the combined administration of mecamylamine and atropine. Antagonizing nAChR and mAChR also prevented the inward current and the mPSC frequency decrease evoked by 5 Hz stimuli. The results confirmed that endogenously released ACh acting on nAChR and mAChR is involved in these events. Most of the septo-hippocampal cholinergic neurons synthesize and cotransmit GABA (Takács et al., 2018). GABA is excitatory to GnRH neurons via GABA_A_ receptors (DeFazio et al., 2002; Moenter and DeFazio, 2005; Farkas et al., 2010), although the sites of its production (Moore et al., 2015; Piet et al., 2018; Silva et al., 2019) have only partially been elucidated. LED illumination of ChR2-tagged cholinergic afferents of GnRH neurons at 0.2 or 5 Hz resulted in a frequency-dependent evoked response. Exclusive GABA release was evoked from cholinergic axons at 0.2 Hz. In contrast, both GABA and ACh were released from axons innervating GnRH neurons at 5 Hz. ACh and GABA cotransmission was earlier demonstrated in the hippocampus (Takács et al., 2018). Kisspeptin and GABA corelease was shown in AVPV neurons innervating GnRH neurons (Piet et al., 2018). Frequency-dependent release was also identified in hypothalamic kisspeptin neurons of mice (Liu et al., 2011; Qiu et al., 2016).

The current study was performed in male mice. Recent data indicate that cholinergic modulation of the GnRH system also occurs in the female (Shostak et al., 2023) where GnRH neurons located in the rostral part of the MS receive even a heavier cholinergic innervation (47%) than those of male mice (26%). Furthermore, the expression of the cholinergic receptor genes (*Chrnb2*, *Chrm4*) in GnRH neurons is influenced by the ovarian cycle (Vastagh et al., 2016).

In addition to the GnRH neurons, the kisspeptin system of the hypothalamus is an expected target of cholinergic modulation that might contribute to the observed activation of the GnRH system. Kisspeptin neurons of the hypothalamus are known to mediate the gonadal hormone feedback effects (Dungan et al., 2006), establish synaptic communication with GnRH neurons (Kalló et al., 2012), and express AChRs (Göcz et al., 2022b,a).

**Figure 14.**
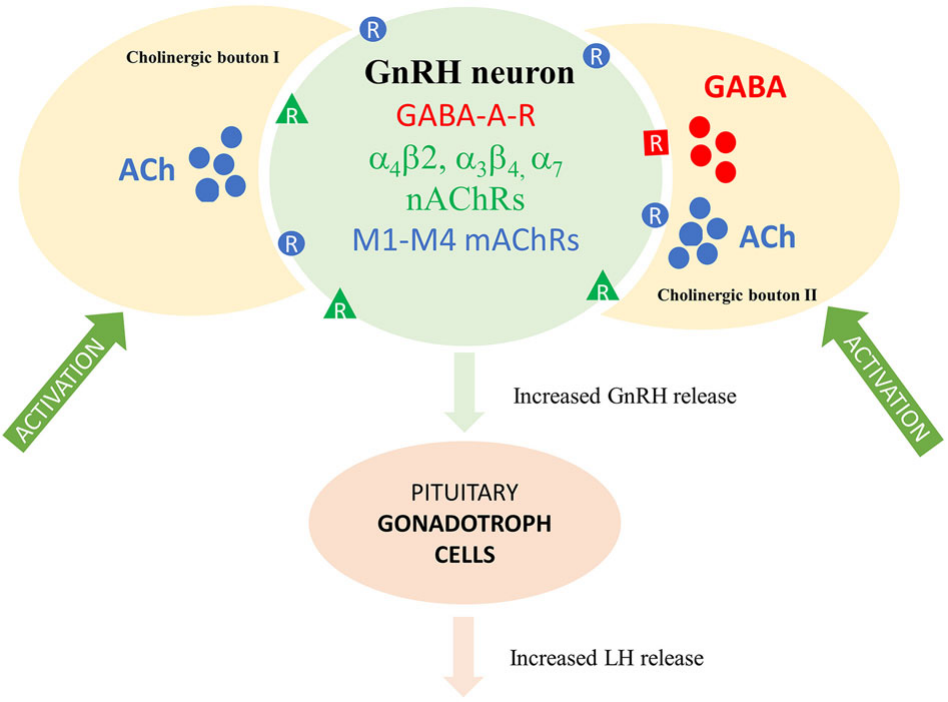
Schematic illustration of cholinergic control of GnRH neurons and the HPG axis in male mice. Cholinergic boutons (I) innervate hypophysiotropic GnRH neurons that express GABA_A_ receptor (GABA_A_-R, red square), three subtypes (α4β2, α3β4, and α7, green triangle) of nAChR, and muscarinic receptors (M1–M4, mAChR, blue oval). The released ACh utilizes both muscarinic and nicotinic receptors in communication with GnRH neurons. At least a subpopulation of cholinergic boutons (II) is capable of transmitting GABA in addition to ACh. Activation of cholinergic boutons increases the firing of GnRH neurons, leading to increased GnRH and concurrent LH release.

In conclusion (Fig. 14), the findings collectively indicate that ACh released from cholinergic afferents, originating from the MS–DBB region, excites GnRH neurons via specific nicotinic and muscarinic receptors in male mice. The activation evokes an augmented GnRH release that, in turn, increases the LH output from the anterior pituitary.
