# Supplementary material for: Cholinergic Control of GnRH Neuron Physiology and Luteinizing Hormone Secretion in Male Mice: Involvement of ACh/GABA Cotransmission
Source: J Neurosci. 2024 Feb 6;44(12):e1780232024. doi: 10.1523/JNEUROSCI.1780-23.2024 (PMC10957212; doi:10.1523/JNEUROSCI.1780-23.2024)
Supplement: Figure 1-1 — Estimated marginal means and contrast of serum LH, LH AUC and peak LH frequency. One-way ANCOVA followed by Tukey's post-test. Download Figure 1-1, DOCX file. [file jneuro-44-e1780232024-s001.docx]

| Extended Data Figure 1-1. Estimated marginal means and contrast of serum LH, LH AUC and peak LH frequency. One-way ANCOVA followed by Tukey's post-test. |
| --- |

|  | group | emmean | SEM | df | lower CL | upper CL | contrast estim. (CNO - VEH) | SEM | df | *t* ratio | *p* value  (vs VEH) |
| --- | --- | --- | --- | --- | --- | --- | --- | --- | --- | --- | --- |
| Mean LH (ng/ml) | VEH | 8.1 | 3.26 | 7 | 0.399 | 15.8 | 13.4 | 5.37 | 7 | 2.491 | 0.0416 |
|  | CNO | 21.5 | 3.26 | 7 | 13.767 | 29.2 |  |  |  |  |  |
| Basal LH (ng/ml) | VEH | 6.84 | 2.03 | 7 | 2.04 | 11.6 | 10 | 3.35 | 7 | 2.988 | 0.0203 |
|  | CNO | 16.86 | 2.03 | 7 | 12.06 | 21.7 |  |  |  |  |  |
| LH AUC (ng/ml * 1 hour) | CNO | 1259 | 189 | 7 | 812 | 1706 | 815 | 309 | 7 | 2.642 | 0.0333 |
|  | VEH | 444 | 189 | 7 | -3 | 891 |  |  |  |  |  |
| LH amplitude (ng/ml) | CNO | 7.36 | 1.91 | 7 | 2.841 | 11.88 | 3.6 | 3.1 | 7 | 1.162 | 0.2834 |
|  | VEH | 3.76 | 1.91 | 7 | -0.764 | 8.27 |  |  |  |  |  |
| LH peak frequency (1/hour) | CNO | 3.58 | 0.47 | 7 | 2.47 | 4.69 | 1.24 | 0.667 | 7 | 1.856 | 0.1058 |
|  | VEH | 4.82 | 0.47 | 7 | 3.71 | 5.93 |  |  |  |  |  |

Mean LH, ANCOVA F(2,7) = 11.38, *p* = 0.00632; basal LH, ANCOVA F(2,7) = 19.21, *p* = 0.00144; LH AUC, ANCOVA F(2,7) = 10.81, *p* = 0.00724; LH amplitude, ANCOVA F(2,7) = 4.283, *p* = 0.061; LH pulse frequency, ANCOVA F(2,7) =1.776, *p* = 0.2378. Confidence level (CL) used: 0.95, emmean: estimated marginal mean.
