# Supplementary material for: Cholinergic Control of GnRH Neuron Physiology and Luteinizing Hormone Secretion in Male Mice: Involvement of ACh/GABA Cotransmission
Source: J Neurosci. 2024 Feb 6;44(12):e1780232024. doi: 10.1523/JNEUROSCI.1780-23.2024 (PMC10957212; doi:10.1523/JNEUROSCI.1780-23.2024)
Supplement: Figure 5-1 — Primer probes used for the quantitative RT-PCR study. Download Figure 5-1, DOCX file. [file jneuro-44-e1780232024-s002.docx]

**Extended Data Fig. 5-1. Primer probes used for the quantitative RT-PCR study.**

|  |  |  |  |  |  |
| --- | --- | --- | --- | --- | --- |
| **Gene symbol** | **Assay ID** | **Interrogated Sequence (RefSeq)** | **Exon boundary** | **Assay location** | **Amplicon  Length (bp)** |
| Chrm1 | Mm00432509_s1 | NM_001112697.1 | 3 | 1566 | 90 |
| Chrm2 | Mm00432509_s2 | NM_203491.3 | 3 | 1828 | 70 |
| Chrm3 | Mm00432509_s3 | NM_033269.4 | 5 | 1030 | 64 |
| Chrm4 | Mm00432509_s4 | NM_007699.2 | 2 | 236 | 53 |
| Chrm5 | Mm00432509_s5 | NM_205783.2 | 1 | 1587 | 87 |
| Chrna2 | Mm00432509_s6 | NM_144803.2 | 6-7 | 1525 | 63 |
| Chrna3 | Mm00432509_s7 | NM_145129.2 | 4-5 | 526 | 67 |
| Chrna4 | Mm00432509_s8 | NM_015730.5 | 4-5 | 496 | 59 |
| Chrna5 | Mm00432509_s9 | NM_176844.4 | 5-6 | 1369 | 73 |
| Chrna6 | Mm00432509_s10 | NM_021369.2 | 1-2 | 327 | 85 |
| Chrna7 | Mm00432509_s11 | NM_007390.3 | 3-4 | 284 | 92 |
| Chrnb1 | Mm00432509_s12 | NM_009601.4 | 3-4 | 299 | 69 |
| Chrnb2 | Mm00432509_s13 | NM_009602.4 | 5-6 | 1564 | 68 |
| Chrnb3 | Mm00432509_s14 | NM_027454.4 | 1-2 | 304 | 61 |
| Chrnb4 | Mm00432509_s15 | NM_148944.4 | 5-6 | 1397 | 68 |
| Chrnd | Mm00432509_s16 | NM_021600.3 | 6-7 | 719 | 68 |
| Chrne | Mm00432509_s17 | NM_009603.1 | 8-9 | 940 | 77 |
| Chrng | Mm00432509_s18 | NM_009604.3 | 6-7 | 615 | 96 |
| Gapdh | Mm00432509_s19 | NM_001289726.1 | 2-3 | 117 | 107 |
| Gnrh1 | Mm00432509_s21 | NM_008145.2 | 2-3 | 240 | 97 |
| Hprt | Mm00432509_s22 | NM_013556.2 | 2-3 | 276 | 131 |
