# Supplementary material for: Cholinergic Control of GnRH Neuron Physiology and Luteinizing Hormone Secretion in Male Mice: Involvement of ACh/GABA Cotransmission
Source: J Neurosci. 2024 Feb 6;44(12):e1780232024. doi: 10.1523/JNEUROSCI.1780-23.2024 (PMC10957212; doi:10.1523/JNEUROSCI.1780-23.2024)
Supplement: Figure 6-1 — Two-way ANOVA and Tukey's post-hoc tests of mPSC frequency data in Fig. 6. Download Figure 6-1, DOCX file. [file jneuro-44-e1780232024-s003.docx]

**Extended data Figure 6-1. Two-way ANOVA and Tukey’s post-hoc tests of mPSC frequency data in Fig. 6.**

mPSC frequency changes between phases significantly.

Frequency data (Hz, mean±SEM):

|  | **ctrl** | **phase I** | **washout** | **N/n** |
| --- | --- | --- | --- | --- |
| **carbachol** | 0.41±0.091 | 0.21±0.050 | 0.41±0.093 | 4/10 |
| **mecamylamine + carbachol** | 0.54±0.16 | 0.26±0.083 | 0.51±0.14 | 3/10 |
| **mecamylamine + atropin + carbachol** | 0.88±0.14 | 0.87±0.11 | 0.89±0.14 | 5/10 |
| **muscarine** | 0.97±0.18 | 0.66±0.14 | 1.0±0.18 | 4/10 |
| **atropin + muscarine** | 0.94±0.19 | 0.91±0.20 | 0.91±0.17 | 5/10 |

N/n= number of animals/number of measured cells

ANOVA table:

|  | **DF** | **F (DFn, DFd)** | **P value** |
| --- | --- | --- | --- |
| **Interaction** | 8 | F (8, 90) = 3.581 | 0.0012* |
| **Phases Factor** | 2 | F (1.434, 64.55) = 23.45 | 0.0001* |
| **Treatment Factor** | 4 | F (4, 45) = 3.941 | 0.0079* |
| **Subject** | 45 | F (45, 90) = 29.44 | 0.0001* |

DF=degree of freedom

*=p<0.05

Tukey’s post-hoc table:

|  | **P value** |
| --- | --- |
| **carbachol** |  |
| ctrl vs. phase I | 0.0031* |
| ctrl vs. washout | 0.9028* |
| phase I vs. washout | 0.0082* |
| **mecamylamine + carbachol** |  |
| ctrl vs. phase I | 0.0219* |
| ctrl vs. washout | 0.5342 |
| phase I vs. washout | 0.0088* |
| **mecamylamine + atropin + carbachol** |  |
| ctrl vs. phase I | 0.9268 |
| ctrl vs. washout | 0.9268 |
| phase I vs. washout | 0.9268 |
| **muscarine** |  |
| ctrl vs. phase I | 0.0329* |
| ctrl vs. washout | 0.5156 |
| phase I vs. washout | 0.0203* |
| **atropin + muscarine** |  |
| ctrl vs. phase I | 0.8074 |
| ctrl vs. washout | 0.6835 |
| phase I vs. washout | 0.9953 |
