# Supplementary material for: Cholinergic Control of GnRH Neuron Physiology and Luteinizing Hormone Secretion in Male Mice: Involvement of ACh/GABA Cotransmission
Source: J Neurosci. 2024 Feb 6;44(12):e1780232024. doi: 10.1523/JNEUROSCI.1780-23.2024 (PMC10957212; doi:10.1523/JNEUROSCI.1780-23.2024)
Supplement: Figure 7-1 — Two-way ANOVA and Tukey’s post-hoc tests of firing rate data in Fig. 7. Download Figure 7-1, DOCX file. [file jneuro-44-e1780232024-s004.docx]

**Extended data Figure 7-1. Two-way ANOVA and Tukey’s post-hoc tests of firing rate data in Fig. 7.**

Firing rate changes between phases significantly.

Firing rate data (Hz, mean±SEM):

|  | **ctrl** | **phase I** | **phase II** | **phase III** | **washout** | **N/n** |
| --- | --- | --- | --- | --- | --- | --- |
| **ACh** | 0.86±0.22 | 3.0±0.55 | 2.1±0.31 | 0.28±0.080 | 0.84±0.19 | 4/8 |
| **carbachol** | 1.0±0.20 | 1.8±0.26 | 1.5±0.18 | 0.23±0.053 | 1.1±0.19 | 4/8 |
| **picro+kynu+carbachol** | 1.1±0.13 | 2.1±0.24 | 1.2±0.09 | 1.1±0.11 | 1.2±0.12 | 3/8 |
| **mecamylamine + carbachol** | 2.9±0.64 | 2.9±0.65 | 4.1±0.71 | 0.44±0.069 | 2.3±0.42 | 3/8 |
| **atropin + carbachol** | 0.96±0.21 | 2.2±0.47 | 1.0±0.20 | 0.86±0.20 | 0.97±0.18 | 4/8 |

N/n= number of animals/number of measured cells

ANOVA table:

|  | **DF** | **F (DFn, DFd)** | **P value** |
| --- | --- | --- | --- |
| **Interaction** | 16 | F (16, 140) = 9.151 | 0.0001* |
| **Phases Factor** | 4 | F (1.909, 66.82) = 62.19 | 0.0001* |
| **Treatment Factor** | 4 | F (4, 35) = 4.524 | 0.0047* |
| **Subject** | 35 | F (35, 140) = 9.588 | 0.0001* |

Tukey’s post-hoc table:

|  | **P value** |
| --- | --- |
| **ACh** |  |
| Ctrl vs. Phase I | 0.0043* |
| Ctrl vs. Phase II | 0.0030* |
| Ctrl vs. Phase III | 0.0457* |
| Ctrl vs. washout | 0.9992 |
| Phase I vs. Phase II | 0.1253 |
| Phase I vs. Phase III | 0.0067* |
| Phase I vs. washout | 0.0063* |
| Phase II vs. Phase III | 0.0018* |
| Phase II vs. washout | 0.0029* |
| Phase III vs. washout | 0.0250* |
| **carbachol** |  |
| Ctrl vs. Phase I | 0.0022* |
| Ctrl vs. Phase II | 0.0123* |
| Ctrl vs. Phase III | 0.0478* |
| Ctrl vs. washout | 0.9974 |
| Phase I vs. Phase II | 0.0638 |
| Phase I vs. Phase III | 0.0044* |
| Phase I vs. washout | 0.0099* |
| Phase II vs. Phase III | 0.0022* |
| Phase II vs. washout | 0.0444* |
| Phase III vs. washout | 0.0321* |
| **picro + kynu + carbachol** |  |
| Ctrl vs. Phase I | 0.0031* |
| Ctrl vs. Phase II | 0.6995 |
| Ctrl vs. Phase III | 0.9854 |
| Ctrl vs. washout | 0.5403 |
| Phase I vs. Phase II | 0.0082* |
| Phase I vs. Phase III | 0.0067* |
| Phase I vs. washout | 0.0041* |
| Phase II vs. Phase III | 0.8691 |
| Phase II vs. washout | 0.9866 |
| Phase III vs. washout | 0.7925 |
| **mecamylamine + carbachol** |  |
| Ctrl vs. Phase I | 0.9839 |
| Ctrl vs. Phase II | 0.0061* |
| Ctrl vs. Phase III | 0.0484* |
| Ctrl vs. washout | 0.3878 |
| Phase I vs. Phase II | 0.0120* |
| Phase I vs. Phase III | 0.0493* |
| Phase I vs. washout | 0.4068 |
| Phase II vs. Phase III | 0.0120* |
| Phase II vs. washout | 0.0042* |
| Phase III vs. washout | 0.0310* |
| **atropine + carbachol** |  |
| Ctrl vs. Phase I | 0.0234* |
| Ctrl vs. Phase II | 0.9232 |
| Ctrl vs. Phase III | 0.7554 |
| Ctrl vs. washout | 0.9998 |
| Phase I vs. Phase II | 0.0393* |
| Phase I vs. Phase III | 0.0179* |
| Phase I vs. washout | 0.0450* |
| Phase II vs. Phase III | 0.5417 |
| Phase II vs. washout | 0.9684 |
| Phase III vs. washout | 0.8685 |
