# Supplementary material for: Cholinergic Control of GnRH Neuron Physiology and Luteinizing Hormone Secretion in Male Mice: Involvement of ACh/GABA Cotransmission
Source: J Neurosci. 2024 Feb 6;44(12):e1780232024. doi: 10.1523/JNEUROSCI.1780-23.2024 (PMC10957212; doi:10.1523/JNEUROSCI.1780-23.2024)
Supplement: Figure 8-1 — One-way ANOVA of amplitudes of the inward currents in Fig. 8. Download Figure 8-1, DOCX file. [file jneuro-44-e1780232024-s005.docx]

**Extended data Figure 8-1. One-way ANOVA of amplitudes of the inward currents in Fig. 8.**

Amplitude of the nAChR agonists-induced inward current changes significantly upon application of the various antagonists

| **F** | **df** | **p** |  | **p (Tukey's test)** |
| --- | --- | --- | --- | --- |
|  |  |  | **Fig. 8a-d.** |  |
| 0.076 | 26 | 0.9265 | "nicotine 1st" vs. "nicotine 2nd" | 0.9870 |
|  |  |  | "nicotine 1st" vs. "picro-kynu+nicotine" | 0.9701 |
|  |  |  | "nicotine 2nd" vs. " picro-kynu+nicotine " | 0.9204 |
|  |  |  | **Fig. 8e-h.** |  |
| 15.5 | 26 | 0.0037 | "nicotine" vs. "DHBE+nicotine" | 0.025 |
|  |  |  | "nicotine" vs. "DHBE+cono+nicotine" | 0.0068 |
|  |  |  | "DHBE+nicotine" vs. "DHBE+cono+nicotine" | 0.0005 |
|  |  |  | **Fig. 8i-l.** |  |
| 12.31 | 26 | 0.0002 | “nicotine” vs. “cono+nicotine” | 0.0064 |
|  |  |  | “nicotine” vs. “cono+DHBE+nicotine” | 0.0002 |
|  |  |  | “cono+nicotine” vs. “cono+DHBE+nicotine” | 0.0431 |
|  |  |  | **Fig. 8m-p.** |  |
| 12.17 | 26 | 0.0002 | "RJR" vs. "DHBE+RJR" | 0.0002 |
|  |  |  | "RJR" vs. "PNU" | 0.0364 |
|  |  |  | "DHBE+RJR" vs. "PNU" | 0.114 |
