# Supplementary material for: Cholinergic Control of GnRH Neuron Physiology and Luteinizing Hormone Secretion in Male Mice: Involvement of ACh/GABA Cotransmission
Source: J Neurosci. 2024 Feb 6;44(12):e1780232024. doi: 10.1523/JNEUROSCI.1780-23.2024 (PMC10957212; doi:10.1523/JNEUROSCI.1780-23.2024)
Supplement: Figure 8-2 — Two-tailed Student’s t-test of amplitudes of inward currents in Fig. 8. Download Figure 8-2, DOCX file. [file jneuro-44-e1780232024-s006.docx]

**Extended data Figure 8-2. Two-tailed Student’s t-test of amplitudes of inward currents in Fig. 8.**

Amplitude of the induced inward current is significant.

| **Figure 8a-d** | **Current amplitude (pA)** | **N/n** | **df** | **t** | **p** |
| --- | --- | --- | --- | --- | --- |
| nicotine 1st | -45.2 ± 8.26 | 3/9 | 8 | 5.47 | 0.0006 |
| nicotine 2nd | -46.8 ± 9.12 | 4/9 | 8 | 5.132 | 0.0009 |
| picro+kynu+nicotine | -42.6 ± 4.69 | 4/9 | 8 | 9.093 | 0.0001 |
| **Figure 8e-h** | **Current amplitude (pA)** | **N/n** | **df** | **t** | **p** |
| nicotine | -41.5 ± 9.05 | 3/9 | 8 | 4.59 | 0.0018 |
| DHBE+nicotine | -18.4 ± 3.41 | 3/9 | 8 | 5.39 | 0.0007 |
| DHBE+conotoxin+nicotine | -9.2 ± 2.36 | 3/9 | 8 | 3.909 | 0.0045 |
| **Figure 8i-l** | **Current amplitude (pA)** | **N/n** | **df** | **t** | **p** |
| nicotine | -42.9 ± 7.89 | 4/9 | 8 | 5.444 | 0.0006 |
| conotoxin+nicotine | -19.2 ± 2.83 | 4/9 | 8 | 6.771 | 0.0001 |
| conotoxin+DHBE+nicotine | -9.2 ± 1.74 | 4/9 | 8 | 5.281 | 0.0007 |
| **Figure 8m-p** | **Current amplitude (pA)** | **N/n** | **df** | **t** | **p** |
| RJR | -22.9 ± 4.15 | 4/11 | 10 | 5.512 | 0.0003 |
| RJR+DHBE | -1.2 ± 1.23 | 3/8 | 7 | 0.9918 | 0.3543 |
| PNU | -11.2 ± 1.88 | 3/8 | 7 | 5.93 | 0.0006 |
