# Supplementary material for: Cholinergic Control of GnRH Neuron Physiology and Luteinizing Hormone Secretion in Male Mice: Involvement of ACh/GABA Cotransmission
Source: J Neurosci. 2024 Feb 6;44(12):e1780232024. doi: 10.1523/JNEUROSCI.1780-23.2024 (PMC10957212; doi:10.1523/JNEUROSCI.1780-23.2024)
Supplement: Figure 9-1 — Two-way ANOVA and Tukey’s post-hoc tests of mPSC frequency data in Fig. 9. Download Figure 9-1, DOCX file. [file jneuro-44-e1780232024-s007.docx]

**Extended data Figure 9-1. Two-way ANOVA and Tukey’s post-hoc tests of mPSC frequency data in Fig. 9.**

mPSC frequency increases/decreases significantly upon muscarine application dependently from the various cocktails of mAChR inhibitors.

Frequency data (Hz, mean±SEM):

|  | **ctrl** | **inhibitor cocktail** | **cocktail+muscarine** | **N/n** |
| --- | --- | --- | --- | --- |
| **tropi+methoct** | 1.8±0.10 | 2.3±0.23 | 3.1±0.39 | 3/10 |
| **dari+piren** | 1.6±0.12 | 1.3±0.11 | 0.99±0.10 | 4/10 |
| **THL+tropi+methoct** | 1.3±0.22 | 1.4±0.24 | 1.5±0.28 | 4/10 |
| **THL+ dari+piren** | 1.3±0.20 | 1.3±0.20 | 1.4±0.20 | 4/10 |

N/n= number of animals/number of measured cells

ANOVA table:

|  | **DF** | **F (DFn, DFd)** | **P value** |
| --- | --- | --- | --- |
| **Interaction** | 6 | F (6, 72) = 16.08 | 0.0001* |
| **Phases Factor** | 2 | F (1.473, 53.02) = 5.649 | 0.0114* |
| **Treatment Factor** | 3 | F (3, 36) = 7.036 | 0.0008* |
| **Subject** | 36 | F (36, 72) = 10.54 | 0.0001* |

Tukey’s post-hoc table:

|  | **P value** |
| --- | --- |
| **tropi+methoct cocktail** |  |
| ctrl vs. cocktail | 0.0351* |
| ctrl vs. cocktail+muscarine | 0.0048* |
| cocktail vs. cocktail+muscarine | 0.0407* |
| **dari+piren cocktail** |  |
| ctrl vs. cocktail | 0.0007* |
| ctrl vs. cocktail+muscarine | 0.0001* |
| cocktail vs. cocktail+muscarine | 0.0012* |
| **THL+tropi+methoct cocktail** |  |
| ctrl vs. cocktail | 0.1665 |
| ctrl vs. cocktail+muscarine | 0.1639 |
| cocktail vs. cocktail+muscarine | 0.2137 |
| **THL+dari+piren cocktail** |  |
| ctrl vs. cocktail | 0.9976 |
| ctrl vs. cocktail+muscarine | 0.9159 |
| cocktail vs. cocktail+muscarine | 0.8410 |
