# Supplementary material for: Cholinergic Control of GnRH Neuron Physiology and Luteinizing Hormone Secretion in Male Mice: Involvement of ACh/GABA Cotransmission
Source: J Neurosci. 2024 Feb 6;44(12):e1780232024. doi: 10.1523/JNEUROSCI.1780-23.2024 (PMC10957212; doi:10.1523/JNEUROSCI.1780-23.2024)
Supplement: Figure 10-1 — Two-way ANOVA and Tukey’s post-hoc tests of firing rate data in Fig. 10. Download Figure 10-1, DOCX file. [file jneuro-44-e1780232024-s008.docx]

**Extended data Figure 10-1. Two-way ANOVA and Tukey’s post-hoc tests of firing rate data in Fig. 10.**

Firing rate changes between phases significantly but dependently from the mAChR inhibitors applied.

Firing rate data (Hz, mean±SEM):

|  | **ctrl** | **phase I** | **phase II** | **washout** | **N/n** |
| --- | --- | --- | --- | --- | --- |
| **muscarine** | 1.5±0.35 | 2.5±0.62 | 0.85±0.24 | 1.5±0.32 | 3/9 |
| **tropi + muscarine** | 2.4±0.51 | 3.9±0.94 | 1.8±0.39 | 2.4±0.52 | 6/9 |
| **methoct +muscarine** | 2.4±0.38 | 3.5±0.56 | 2.0±0.33 | 2.3±0.36 | 4/9 |
| **methoct + tropi+ muscarine** | 1.2±0.17 | 1.6±0.18 | 1.5±0.18 | 1.4±0.17 | 4/9 |
| **piren + methoct + tropi+ muscarine** | 0.91±0.17 | 1.2±0.21 | 0.95±0.19 | 0.87±0.15 | 4/9 |
| **dari + methoct + tropi+ muscarine** | 1.2±0.16 | 1.4±0.14 | 1.5±0.19 | 1.3±0.15 | 4/9 |
| **dari + piren + methoct + tropi+ muscarine** | 1.7±0.34 | 1.8±0.32 | 1.7±0.33 | 1.9±0.35 | 3/9 |

N/n= number of animals/number of measured cells

ANOVA table:

|  | **DF** | **F (DFn, DFd)** | **P value** |
| --- | --- | --- | --- |
| **Interaction** | 18 | F (18, 168) = 7.885 | 0.0001* |
| **Phases Factor** | 3 | F (1.285, 71.94) = 43.18 | 0.0001* |
| **Treatment Factor** | 6 | F (6, 56) = 3.226 | 0.0086* |
| **Subject** | 56 | F (56, 168) = 25.17 | 0.0001* |

Tukey’s post-hoc table:

|  | **P value** |
| --- | --- |
| **Muscarine** |  |
| ctrl vs. phase I | 0.0266* |
| ctrl vs. phase II | 0.0381* |
| ctrl vs. washout | 0.9983 |
| phase I vs. phase II | 0.0167* |
| phase I vs. washout | 0.0476* |
| phase II vs. washout | 0.0349* |
| **tropi + muscarine** |  |
| ctrl vs. phase I | 0.0479* |
| ctrl vs. phase II | 0.0150* |
| ctrl vs. washout | 0.9703 |
| phase I vs. phase II | 0.0311* |
| phase I vs. washout | 0.0406* |
| phase II vs. washout | 0.0428* |
| **methoct +muscarine** |  |
| ctrl vs. phase I | 0.0060* |
| ctrl vs. phase II | 0.0183* |
| ctrl vs. washout | 0.3927 |
| phase I vs. phase II | 0.0014* |
| phase I vs. washout | 0.0081* |
| phase II vs. washout | 0.0416* |
| **methoct + tropi+ muscarine** |  |
| ctrl vs. phase I | 0.0023* |
| ctrl vs. phase II | 0.0046* |
| ctrl vs. washout | 0.0213* |
| phase I vs. phase II | 0.2076 |
| phase I vs. washout | 0.0763 |
| phase II vs. washout | 0.2425 |
| **piren + methoct + tropi+ muscarine** |  |
| ctrl vs. phase I | 0.0045* |
| ctrl vs. phase II | 0.6144 |
| ctrl vs. washout | 0.3919 |
| phase I vs. phase II | 0.0336* |
| phase I vs. washout | 0.0129* |
| phase II vs. washout | 0.2716 |
| **dari + methoct + tropi+ muscarine** |  |
| ctrl vs. phase I | 0.0009* |
| ctrl vs. phase II | 0.0405* |
| ctrl vs. washout | 0.0194* |
| phase I vs. phase II | 0.5930 |
| phase I vs. washout | 0.9443 |
| phase II vs. washout | 0.2240 |
| **dari + piren + methoct + tropi+ muscarine** |  |
| ctrl vs. phase I | 0.9755 |
| ctrl vs. phase II | 0.9999 |
| ctrl vs. washout | 0.2551 |
| phase I vs. phase II | 0.9917 |
| phase I vs. washout | 0.9639 |
| phase II vs. washout | 0.4809 |
