# Supplementary material for: Cholinergic Control of GnRH Neuron Physiology and Luteinizing Hormone Secretion in Male Mice: Involvement of ACh/GABA Cotransmission
Source: J Neurosci. 2024 Feb 6;44(12):e1780232024. doi: 10.1523/JNEUROSCI.1780-23.2024 (PMC10957212; doi:10.1523/JNEUROSCI.1780-23.2024)
Supplement: Figure 11-1 — Two-way ANOVA and Tukey’s post-hoc tests of firing rate data in Fig. 11. Download Figure 11-1, DOCX file. [file jneuro-44-e1780232024-s009.docx]

**Extended data Figure 11-1. Two-way ANOVA and Tukey’s post-hoc tests of firing rate data in Fig. 11.**

Firing rate changes between phases significantly.

Firing rate data (Hz, mean±SEM):

|  | **ctrl** | **phase I** | **phase II** | **washout** | **N/n** |
| --- | --- | --- | --- | --- | --- |
| **LED** | 1.5±0.35 | 2.5±0.62 | 0.85±0.24 | 1.5±0.32 | 3/15 |
| **atropine + mecamylamine + LED** | 2.4±0.51 | 3.9±0.94 | 1.8±0.39 | 2.4±0.52 | 6/15 |

N/n= number of animals/number of measured cells

ANOVA table of firing rate:

|  | **DF** | **F (DFn, DFd)** | **P value** |
| --- | --- | --- | --- |
| **Interaction** | 3 | F (3, 63) = 6.901 | 0.0004* |
| **Phases Factor** | 3 | F (1.664, 34.94) = 10.76 | 0.0005* |
| **Treatment Factor** | 1 | F (1, 21) = 4.454 | 0.0470* |
| **Subject** | 21 | F (21, 63) = 56.23 | 0.0001* |

Tukey’s post-hoc table of firing rate:

|  | **P value** |
| --- | --- |
| **LED** |  |
| ctrl vs. phase I | 0.0190* |
| ctrl vs. phase II | 0.0040* |
| ctrl vs. washout | 0.8456 |
| phase I vs. phase II | 0.0001* |
| phase I vs. washout | 0.0263* |
| phase II vs. washout | 0.0042* |
| **atropine + mecamylamine + LED** |  |
| ctrl vs. phase I | 0.7894 |
| ctrl vs. phase II | 0.9241 |
| ctrl vs. washout | 0.4968 |
| phase I vs. phase II | 0.9044 |
| phase I vs. washout | 0.6000 |
| phase II vs. washout | 0.7141 |
