# Supplementary material for: Cholinergic Control of GnRH Neuron Physiology and Luteinizing Hormone Secretion in Male Mice: Involvement of ACh/GABA Cotransmission
Source: J Neurosci. 2024 Feb 6;44(12):e1780232024. doi: 10.1523/JNEUROSCI.1780-23.2024 (PMC10957212; doi:10.1523/JNEUROSCI.1780-23.2024)
Supplement: Figure 11-2 — Two-way ANOVA and Tukey’s post-hoc tests of mPSC frequency data in Fig. 11. Download Figure 11-2, DOCX file. [file jneuro-44-e1780232024-s010.docx]

**Extended data Figure 11-2. Two-way ANOVA and Tukey’s post-hoc tests of mPSC frequency data in Fig. 11.**

mPSC frequency changes between phases significantly.

mPSC frequency data (Hz, mean±SEM):

|  | **ctrl** | **phase I** | **washout** | **N/n** |
| --- | --- | --- | --- | --- |
| **LED** | 1.9±0.48 | 1.0±0.20 | 1.8±0.44 | 10/27 |
| **atropine + mecamylamine + LED** | 1.6±0.29 | 1.9±0.40 | 1.7±0.31 | 4/8 |

N/n= number of animals/number of measured cells

ANOVA table of mPSC frequency:

|  | **DF** | **F (DFn, DFd)** | **P value** |
| --- | --- | --- | --- |
| **Interaction** | 2 | F (2, 66) = 3.198 | 0.0472* |
| **Row Factor** | 2 | F (1.027, 33.89) = 0.7768 | 0.3876 |
| **Column Factor** | 1 | F (1, 33) = 0.04377 | 0.8356 |
| **Subject** | 33 | F (33, 66) = 11.73 | 0.0001* |

Tukey’s post-hoc table of mPSC frequency:

|  | **P value** |
| --- | --- |
| **LED** |  |
| ctrl vs. phase I | 0.0454* |
| ctrl vs. washout | 0.3398 |
| phase I vs. washout | 0.0426* |
| **atropine + mecamylamine + LED** |  |
| ctrl vs. phase I | 0.5429 |
| ctrl vs. washout | 0.7856 |
| phase I vs. washout | 0.4683 |
